# Structure-activity correlations for peptaibols obtained from clade Longibrachiatum of *Trichoderma*: A combined experimental and computational approach

**DOI:** 10.1016/j.csbj.2023.02.046

**Published:** 2023-02-24

**Authors:** Dóra Balázs, Tamás Marik, András Szekeres, Csaba Vágvölgyi, László Kredics, Chetna Tyagi

**Affiliations:** a Department of Microbiology, Faculty of Science and Informatics, University of Szeged, Közép fasor 52, H-6726 Szeged, Hungary; b Doctoral School of Biology, Faculty of Science and Informatics, University of Szeged, Közép fasor 52, H-6726 Szeged, Hungary

**Keywords:** *Trichoderma*, Peptaibols, Molecular dynamics simulations, Peptide folding, Structure-activity relationship (SAR)

## Abstract

Integrated disease management and plant protection have been discussed with much fervor in the past decade due to the rising environmental concerns of using industrially produced pesticides. Members of the genus *Trichoderma* are a subject of considerable research today due to their several properties as biocontrol agents. In our study, the peptaibol production of *Trichoderma longibrachiatum* SZMC 1775, *T. longibrachiatum* f. *bissettii* SZMC 12546, *T. reesei* SZMC 22616, *T. reesei* SZMC 22614, *T. saturnisporum* SZMC 22606 and *T. effusum* SZMC 22611 were investigated to elucidate structure-activity relationships (SARs) between the properties of peptaibols and their 3D structures. The effects of peptaibol mixtures obtained from every *Trichoderma* strain were examined against nine commonly known bacteria. The lowest minimum inhibitory concentrations (MIC, mg ml^−1^) were exerted by *T. longibrachiatum* f. *bissettii* SZMC 12546 against Gram-positive bacteria, which was also able to inhibit the plant pathogenic Gram-negative *Rhizobium radiobacter*. Accelerated molecular dynamics (aMD) simulations were performed in aqueous solvent to explore the folding dynamics of 12 selected peptaibol sequences. The most characteristic difference between the peptaibols from group A and B relies in the ‘Gly-Leu-Aib-Pro’ and ‘Gly-Aib-Aib-Pro’ motifs (‘Aib’ stands for α-aminoisobutyric acid), which imparted a significant effect on the folding dynamics in water and might be correlated with their expressed bioactivity. In our aMD simulation experiments, Group A peptaibols showed more restricted folding dynamics with well-folded helical conformations as the most stable representative structures. This structural stability and dynamics may contribute to their bioactivity against the selected bacterial species.

## Graphical Abstract

**Figure:**
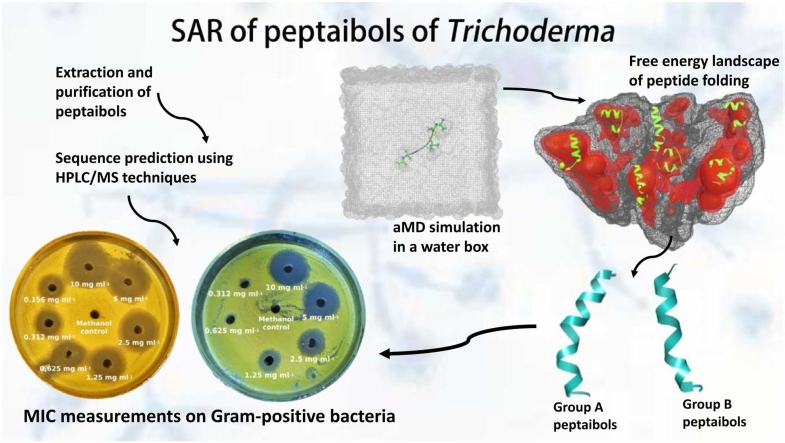

## 1 Introduction

Today more than 415 species from the genus *Trichoderma* are known, and the attention is drawn to certain species owing to their biocontrol influence on phytopathogenic microorganisms and positive effects on plant growth [70]. In nature, *Trichoderma* species exhibit antagonistic effects on other microorganisms in the environment with their increased growth and the production of bioactive secondary metabolites in order to gain their own ground [35]. These metabolites inhibit the growth of other fungi or bacteria through various pathways where usually the primary target is the membrane [16]. Due to their several favorable properties, the members of the genus are used widely in the fields of industry and agriculture. Their mycoparasitic, saprophytic and symbiotic abilities can provide protection against plant pathogens and also support plant development [17], [35], [46]. Clade Longibrachiatum within the genus *Trichoderma* forms a smaller, monophyletic group which is phylogenetically further away from the other four sections (*Pachybasium*, *Trichoderma*, *Saturnisporum* and *Hypocreanum*) of the genus [3]. Within the clade, there are extremely diverse species that not only have advantageous properties but may also have a clinically significant role [30], [34], [48].

The important role of *Trichoderma* strains is partially due to their secondary metabolites, out of which, approximately 80% belong to the peptaibiotics class [57]. One large group of peptaibiotics is the peptaibols which are characterized by the generally acetylated N-terminus, the amino-alcohol group like isoleucinol and phenylalaninol on the C-terminus, and the presence of non-proteinogenic amino acids, such as α-aminoisobutyric acid (Aib), hydroxyproline or D-isovaline [62]. The high degree of variability is due to their synthesis carried out by the non-ribosomal peptide synthetases (NRPSs) with modular structure, while the addition of individual amino acid substitutions, deletions and the presence of non-proteinogenic amino acids also increase the number of possible variations [60]. Peptaibols usually form helical, slightly amphipathic structures that results in a hydrophobic and a hydrophilic face on the folded conformation.

The amphipathic nature of long (18–20 residue) peptaibols helps their self-assembly, which often results in ion-channel formation in phospholipid membranes through which ions and water can leave the lumen, thereby causing cell death [18], [9]. The hydrophilic face lines inside of the lumen while the hydrophobic side faces the transmembrane region. A few others may instead show a carpet-like detergent effect on the surface of bilayer membranes [20]. The conformation and channel-forming properties of each peptide sequence are determined by the physicochemical properties of the constituting amino acids. It is known that the high Aib content in sequences leads to the helical conformation of peptaibols [53], [58]. The increased Aib content, especially in the first and second positions, is also responsible for the potent resistance of peptaibols to the effect of proteolytic enzymes [68]. However, channel formation is also influenced by other factors, for example, the higher peptide concentration is an important determining factor as seen in the case of alamethicin [31], [32]. Shorter peptaibols are also able to form ion channels in the membrane. However, in this case, specific interactions may occur between the N- and C- terminals and the phospholipid head groups, thereby, causing membrane thinning [5]. Analogues created by the modification of natural peptaibols may help to understand the effect of the amount and position of each amino acid in the sequence. Recently, De Zotti et al. [15] created and tested the efficacy of water-soluble analogues of the peptaibol trichogin, which had an increased antifungal activity against three common fungal plant pathogens. Apart from direct antagonism against various bacteria, peptaibols have also been reported to affect the jasmonate and ethylene response pathways in plants, which leads to an induced systemic resistance (ISR) providing protection against several plant pathogens [21], [29], [47]. Therefore, their characterization from the perspective of integrated pest management may highlight novel, organically produced, and fail-safe ways to fight agriculturally relevant pathogens.

In this study, we compare the results of bioactivity experiments against commonly known phytopathogenic bacteria with the structural understanding of various peptaibol sequences obtained through computational modelling. The development of computational methods facilitates the study of the relationships between conformational and bioactivity properties of peptaibols. The folding process of peptaibols can be examined using a relatively new enhanced sampling technique: accelerated molecular dynamics (aMD). During the aMD process, the potential energy surface can be modified by applying a non-negative boost potential, thereby, reducing the folding time of peptides [28]. The isolated peptaibol mixtures produced by each *Trichoderma* strain were used to determine the MIC values, but only the most produced sequences and their analogous primary sequences from the other group were selected for further *in silico* experiments. The knowledge of correlations between biological activity and the characteristics of peptide conformations can promote the selection of *Trichoderma* strains that produce highly bioactive peptaibols. Furthermore, the peptaibol extracts may prove to be of great importance in agriculture and can promote the development of chemical-free plant protection in the future.

## 2 Materials and methods

### 2.1 Microorganisms

**Fig. 1:**
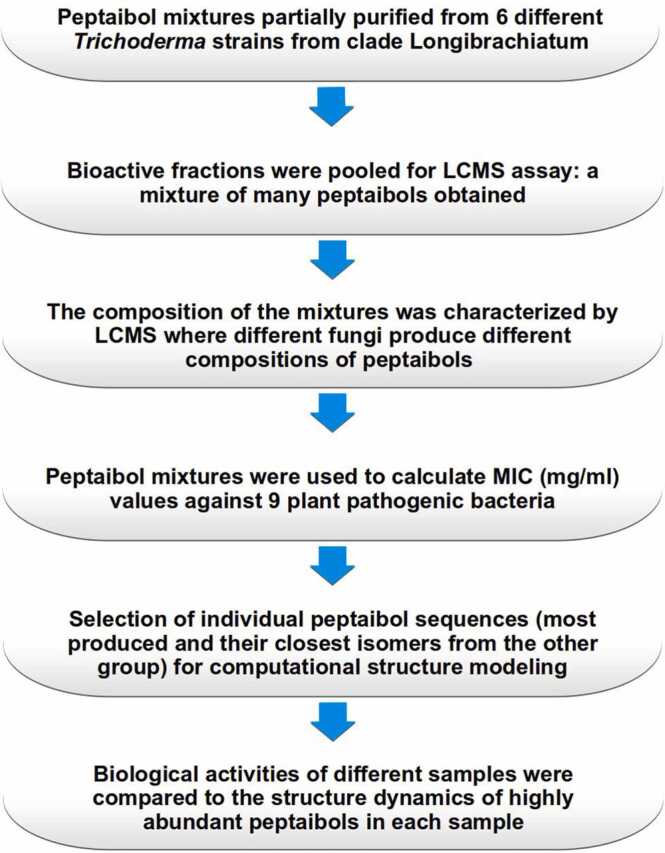
A flowchart of the methodology used in this work.

Six *Trichoderma* strains from clade Longibrachiatum of the genus were selected from the Szeged Microbiology Collection, Szeged, Hungary (SZMC; www.szmc.hu) for peptaibol production (Table 1). The MIC values of the peptaibol extracts were determined on 9 bacterial strains selected from the SZMC (Table 2). A flowchart of the methodology used in this research has been added as Fig. 1.

**Table 1:** *Trichoderma* strains used in this study.

Species	SZMC identifier	Other identifier	Isolation		Reference
*T. effusum*	22611	TUCIM 254		Soil, Himalaya, India	[4]
*T. longibrachiatum*	1775	CECT 2937		Antarctica	[37]
*T. longibrachiatum* f. *bissettii*	12546	UAMH 7956		Clinical isolate, bone marrow transplant patient, lung, liver, bone wall	[55]
*T. reesei*	22614	TUCIM 917, QM6a		Canvas of US army, Solomon Islands	[54]
*T. reesei*	22616	QM9414		Mutant of QM9123 (which is mutant of QM6a)	[36]
*T. saturnisporum*	22606	TUCIM 1267		Italy	[59]

**Table 2:** Bacterial strains used for the minimum inhibitory concentration (MIC) tests in this study.

	Plant pathogenic strains	SZMC identifier	Diseases	Reference
Gram-positive	*Clavibacter michiganensis*	0016	Ring rot of potato	[1]
	*Rhodococcus fascians*	21247 MP89	Leafy gall	[27]
Gram-negative	*Rhizobium radiobacter*	21407	Crown gall disease	[12]
	*Xanthomonas campestris*	6182	Black rot disease	[67]
	*Erwinia amylovora*	21402 K21	Fire blight	[72]
	*Pantoea ananatis*	6205 J	Leaf blotches and spots, die‐back, and stalk, fruit and bulb rot	[11]
	**Additional bacterial strains**	**SZMC identifier**		
Gram-positive	*Micrococcus luteus*	6207		
	*Bacillus cereus*	23292		
Gram-negative	*Escherichia coli*	0582

### 2.2 Cultivation and isolation of peptaibol mixtures

The *Trichoderma* strains were cultured on large glass plates (40 × 40 cm) for the large-scale production of peptaibols as described by Marik et al. [43]. The extracted peptaibol samples were further isolated from the impurities with Flash Chromatograph (CombyFlash EZ Prep UV–VIS, Teledyne Isco Inc. Lincoln, NE, USA). The cartridge (CombyFlash EZ Prep) was filled with 50 cm^3^ silica (30–40 µm) and 1.5 g crude peptaibol extract. The flow rate was set to 35 ml min^−1^. Solvent A was chloroform and solvent B was methanol (gradient solvent B: 0%, 0 min; 0%, 5 min; 100%, 15 min; 100% 18 min). The whole eluent was collected into fractions, from the beginning till the end of run, continuously into collector tubes (18 × 180 ml, 10 ml) during the whole separation. The fractions were evaporated and dissolved in methanol (10 mg ml^−1^). Each fraction was tested on *M. luteus* and the active fractions which showed inhibition zone larger than 7 mm in diameter were pooled into a Falcon tube [42]. For the MIC tests, the peptaibol extracts were diluted in methanol using two-fold dilution from 10 mg ml^−1^ to 0.156 mg ml^−1^ concentrations and stored at − 20 °C until further use.

### 2.3 Peptaibol analysis

Peptaibol production of the same *Trichoderma* strains was previously determined by HPLC-ESI-MS and the peptaibols were divided into groups A and B based on the characteristic amino acid composition of the sequences [44]. The composition of peptaibol mixtures obtained during the current HPLC-MS analysis was compared with the results described in the previous study, which showed the same peptaibol composition produced by the *Trichoderma* species (Supplementary Figure 1). The semi-quantitative analysis was done using HPLC-MS as described by van Bohemen et al. [65]. Due to this, the LC–MS quantifications of peptaibols have been performed using alamethicin (Sigma-Aldrich A-4665, Hungary) as standard. Currently, where quantifications were done in the literature, all 18–20 residue peptaibol subgroups could be quantified using only this standard containing a mixture of four 20-residue peptaibols. Thus, the following procedures were used: six concentration levels were prepared of the alamethicin standard (0.1; 0.25; 0.5; 0.75; 1.0 and 1.25 µg ml^−1^) which were dissolved in HPLC quality methanol. Calibration curves were prepared based on the injected quantity of alamethicin standard and the peak area of all y_7_-ion fragments originated from the 20-residue alamethicin standard peptaibols appearing on the extracted chromatograms. Measured quantities of the partially purified peptaibol mixtures were introduced into HPLC-MS and the results were compared to the alamethicin calibration curve and the ratio of the peptaibol composition was calculated. The analysis indicates that the isolated fractions contain peptaibols at high purity (at 99% as the impurities leave the column in the first minutes and later during the run and the eluents contain only the elements of peptaibol mixtures sequences).

### 2.4 Peptaibol bioactivity and MIC test

The partially purified peptaibol mixtures produced by each strain (classified into group A and B) were used to define their MIC values. The MIC values were determined on plates by agar well diffusion assays using methanol as a control. Bacteria were preincubated for two days on 2% LB (Luria Bertani) agar at 28 °C. The 10^5^ cell density for the inoculation used for the tests was determined by spectrophotometer (BioTek Synergy HTX Multi-Mode Reader, Agilent, USA) at OD_620_. Petri plates were filled with 2% LB agar, furthermore, the bacteria were inoculated into 5 ml 1% LB agar, which was applied to the surface of the 2% LB agar. Seven holes (5 mm in diameter) were bored into the agar with sterile cork borer and filled with 40 µl of the peptaibol extracts in different dilutions. After two days of incubation at 28 °C, the inhibition zones were measured, and the MIC values were determined based on the inhibition zones detected at the lowest inhibitory concentrations. The plates were photographed using a Coolpix S2600 digital camera (Nikon, Tokyo, Japan). Each experiment was carried out in three replicates.

### 2.5 Accelerated molecular dynamics (aMD) simulations of the selected peptaibols

Twelve peptaibol sequences belonging to groups A and B were selected for the aMD simulations. These groups were determined by Marik et al. [44], where group A and B contain Lxx and Aib at position 12 of the amino acid sequence, respectively (Table 3). Group A sequences are homologous, similar, or positionally isomeric to the peptaibol subfamilies of trichobrachins, suzukacillins, trichoaureocins, hyporientalins, trichokonins, trilongins, metanicins, gliodeliquescins, trichosporins, and hypophellins, while group B sequences proved to be homologous, similar or positionally isomeric to the peptaibol subfamilies of paracelsins, saturnisporins, trichocellins, and *Trichoderma citrinoviride* sequences [44]. The sequences contain the non-standard residues Aib and phenylalaninol (Pheol), whose partial charges were calculated using the R.E.D server and creating force field libraries. R.E.D stands for RESP ESP charge derive [19], [66]. RESP (restrained electrostatic potential) was used to calculate the charges with a HF/6–31 G(d) basis set and Gaussian 09 quantum mechanical program interface [25]. For each residue, two conformations, *i.e.*, α helix (Φ = −63.8, Ψ = −38.3) and β sheet or C5 (Φ = −157.2, Ψ = 161.9) were used. The terminal residue Pheol was also parameterized using two molecules, ethyl alcohol and Phe.

**Table 3:** The 12 peptaibol sequences selected from both groups A and B to elucidate structure-activity relationships. The b_13_-ion and y_7_p-ion represent the two ion fragments formed by the break between the Aib and Pro amino acid pair during the process of HPLC-MS. Vxx and Lxx represent Valine/Isovaline and Leucine/Isoleucine amino acids, respectively. All data in this table are based on [44].

Name	b_13_-ion	y_7_p-ion	Retention time	Sequence																				
				R	R1	R2	R3	R4	R5	R6	R7	R8	R9	R10	R11	R12	R13	R14	R15	R16	R17	R18	R19	R20
Pept-A-IVa	1163	774	40.21	Ac	Aib	Ala	Aib	Ala	Aib	**Ala**	Gln	Aib	Vxx	Aib	Gly	**Lxx**	Aib	Pro	Vxx	Aib	Aib	Gln	Gln	Pheol
Pept-B-IXa	1135	774	28.44	Ac	Aib	Ala	Aib	Ala	Aib	**Ala**	Gln	Aib	Vxx	Aib	Gly	**Aib**	Aib	Pro	Vxx	Aib	Aib	Gln	Gln	Pheol
Pept-A-XVIa	1177	774	45.21	Ac	Aib	Ala	Aib	Ala	Aib	Aib	Gln	Aib	Vxx	Aib	Gly	**Lxx**	Aib	Pro	Vxx	Aib	Aib	Gln	Gln	Pheol
Pept-B-XVI	1149	774	31.98	Ac	Aib	Ala	Aib	Ala	Aib	Aib	Gln	Aib	Vxx	Aib	Gly	**Aib**	Aib	Pro	Vxx	Aib	Aib	Gln	Gln	Pheol
Pept-A-XXVb	1191	788	49.72	Ac	Aib	Ala	Aib	Ala	Aib	Aib	Gln	Aib	**Lxx**	Aib	Gly	**Lxx**	Aib	Pro	Vxx	Aib	**Vxx**	Gln	Gln	Pheol
Pept-B-XXVII	1163	788	37.31	Ac	Aib	Ala	Aib	Ala	Aib	Aib	Gln	Aib	**Lxx**	Aib	Gly	**Aib**	Aib	Pro	Vxx	Aib	**Vxx**	Gln	Gln	Pheol
Pept-A-XIXa	1177	775	46.67	Ac	Aib	Ala	Aib	Ala	Aib	Aib	Gln	Aib	Vxx	Aib	Gly	**Lxx**	Aib	Pro	Vxx	Aib	Aib	**Glu**	Gln	Pheol
Pept-B-XIVb	1149	775	31.40	Ac	Aib	Ala	Aib	Ala	Aib	Aib	Gln	Aib	Vxx	Aib	Gly	**Aib**	Aib	Pro	Vxx	Aib	Aib	**Glu**	Gln	Pheol
Pept-A-Via	1163	775	41.46	Ac	Aib	Ala	Aib	Ala	Aib	**Ala**	Gln	Aib	Vxx	Aib	Gly	**Lxx**	Aib	Pro	Vxx	Aib	Aib	**Glu**	Gln	Pheol
Pept-B-XIVa	1149	775	31.36	Ac	Aib	Ala	Aib	Ala	Aib	**Ala**	Gln	Aib	**Lxx**	Aib	Gly	**Aib**	Aib	Pro	Vxx	Aib	Aib	**Glu**	Gln	Pheol
Pept-A-XXIIb	1191	774	48.79	Ac	Aib	Ala	Aib	Ala	Aib	Aib	Gln	Aib	**Lxx**	Aib	Gly	**Lxx**	Aib	Pro	Vxx	Aib	Aib	Gln	Gln	Pheol
Pept-B-XX	1163	774	34.41	Ac	Aib	Ala	Aib	Ala	Aib	Aib	Gln	Aib	**Lxx**	Aib	Gly	**Aib**	Aib	Pro	Vxx	Aib	Aib	Gln	Gln	Pheol

The unfolded peptaibol conformations were prepared by using the ‘tleap’ module of AmberTools18 [7] and solvated in water (TIP3P water model). The characteristics of the peptaibol systems are given in Table 4. Amberff14SB force field was used to prepare the system [7]. It was prepared for aMD in six consecutive steps as described by Tyagi et al. [63]. All simulations were carried out at 300 K temperature, 2 fs time step, and energies and boost information were recorded at every 1000 steps. The electrostatic interactions were calculated using PME (particle mesh Ewald summation) [22], long-range interactions were also calculated with cutoff of 10.0. The temperature scaling was carried out using Langevin thermostat without pressure scaling. The SHAKE algorithm was applied on all bonds involving hydrogen. The GPU (graphics processing unit) machines available through the KIFU-HPC (Governmental Agency for IT Development - High Performance Computing) clusters of Hungary were utilized for all aMD simulations using *pmemd.cuda* implementation of Amber16 [6] The dual boost option with iamd= 3 was used for all simulations. The following values of the coefficients were used to the preparations: a1, a2 = 0.16 kcal/mol; b1, b2 = 4 kcal/mol based on previous studies [52].

**Table 4:** Characteristics of the simulation systems of unfolded peptaibols inserted in a TIP3P water box.

Peptaibol system	TIP_3_P water molecules	Box size (Å)	Volume (Å^3^)
PA6A	6450	70.371 × 56.570 × 63.093	251169.066
PA4A	6451	70.371 × 56.570 × 63.093	251169.066
PA16A	4592	53.760 × 50.559 × 67.301	182927.904
PA19A	4593	53.760 × 50.559 × 67.301	182927.904
PA22B	4591	53.760 × 50.559 × 67.301	182927.904
PA25B	4758	58.580 × 46.735 × 68.610	187839.165
PB9A	5096	58.950 × 50.559 × 65.734	195919.502
PB14A	5093	58.950 × 50.559 × 65.734	195919.502
PB14B	5315	55.908 × 57.504 × 64.481	207300.924
PB16A	5314	55.908 × 57.504 × 64.481	207300.924
PB20	5765	55.908 × 62.921 × 62.946	221426.787
PB27	5765	55.908 × 62.921 × 62.946	221426.787

## 3 Results and discussion

### 3.1 Selection of peptaibols

In our previous study, peptaibols produced by the examined *Trichoderma* strains were divided into group A and group B based on the characteristic amino acid compositions of their sequences [44]. In each case, the peptaibol sequences are 20 amino acid residues in length and characterized by the ‘Ac-Aib-Ala-Aib-Aib’ motif at the N-terminus and the Pheol amino-alcohol group at the C-terminus. All sequences contain Aib residues in a high amount. The presence of the ‘Aib-Pro’ labile amide bond at positions R13-R14 causes the cleavage of the sequences into b- and y-ion fragments during HPLC-ESI-MS investigations in the ion source, which show characteristic peaks on the MS spectra. Furthermore, the protonated molecular ions are usually visible on the spectra (Supplementary Figure 1). The ‘Gln-Aib’ motif is also a characteristic amino acid pair in the R7-R8 positions, which is found in all examined sequences. The amino alcohol group is preceded by the Glu-Gln or Gln-Gln amino acid pair in each case. The main differences between the sequences of group A and group B are the ‘Gly-Leu-Aib-Pro’ and ‘Gly-Aib-Aib-Pro’ motifs at positions R11-R14 [44]. This study is aimed at the investigation of the peptaibol production of clade Longibrachiatum, where opportunistic human pathogens can be found. The clinically relevant *T. longibrachiatum* SZMC 12546, and its environmental pair, *T. longibrachiatum* SZMC 1775 were selected from group A peptaibol-producing strains, while biotechnologically important *T. reesei* strains as well as two further strains: *T. saturnisporum* and *T. effusum* were selected from Group B peptaibol producers.

The peptaibol sequences selected from group A for MD simulation studies account for 87.46% in the total peptaibol production of *T. longibrachiatum* f. *bissettii* SZMC 12546 and 70.73% of *T. longibrachiatum* SZMC 1775 (Table 5). In case of Group B, the peptaibols produced in the highest amounts account for about half of the total peptaibol composition of the *Trichoderma* strains (Table 5). From group A, the Pept-A-IVa sequence is produced in the highest amount, accounting for 63% of the total peptaibol composition of *T. longibrachiatum* SZMC 1775 and about 47% of the total composition of *T. longibrachiatum* SZMC 12546. Its analogous sequence pair from group B is produced in a small amount by *T. reesei* strains SZMC 22616 and SZMC 22614, and not produced by the *T. saturnisporum* and *T. effusum* strains at all. Additional sequences are presented in varying proportions in the production profiles of the species. The Pept-A-XXIIb sequence is missing from the profile of two strains in group A, however, it accounts for between 9% and 16% in the group B peptaibol production. The Pept-B-XXVII sequence is produced in the highest amount by all of the strains in group B. The two strains of *T. reesei* have similar peptaibol compositions but produce the sequences in different amounts. *T. reesei* SZMC 22614 is the original strain from Solomon Islands [54], while SZMC 22616 is its mutant strain [36]. From the peptaibol production of each *Trichoderma* strain, the peptaibol sequences produced in the highest amount and their pairs from the other group which can be characterized by the main amino acid differences at positions R12 were selected for the aMD simulations (Table 2). At all positions, Lxx was predicted to be Leu and Vxx to be Val based on the abundance of the amino acids in this position in the sequences from the offline version of the ’Comprehensive Peptaibiotics Database’ [61]. The online resource of Peptaibiotics Database [49] is unavailable since 2017, therefore, publications since 2017 were searched on PubMed with the keyword „peptaibol”.

**Table 5:** The production of the chosen peptaibol sequences (percentage) from the whole peptaibol production of the *Trichoderma* strains belonging to group A and group B. The values mean the percentage of production of each peptide from the peptaibol mixture produced by each strain, however, the area percentages were considered based on the peak area of each y_7_p-ion fragment.

*Group A*	*T. longibrachiatum* SZMC 1775	*T. longibrachiatum* SZMC 12546	*Group B*	*T. reesei* SZMC 22614	*T. reesei* SZMC 22616	*T. saturnisporum* SZMC 22606	*T. effusum* SZMC 22611
Pept-A-IVa	63.78	47.22	Pept-B-IXa	0.38	3.87	-	-
Pept-A-VIa	0.14	0.35	Pept-B-XIVa	-	-	0.01	0.05
Pept-A-XVIa	32.13	19.00	Pept-B-XVI	0.47	-	-	-
Pept-A-XIXa	0.18	0.41	Pept-B-XIVb	0.03	0.23	-	-
Pept-A-XXIIb	-	-	Pept-B-XX	42.26	14.97	9.67	12.81
Pept-A-XXVb	0.11	-	Pept-B-XXVII	44.50	21.04	40.96	46.88
**Total amount (%)**	**96.34**	**66.98**	**Total amount (%)**	**87.64**	**40.11**	**50.64**	**59.74**

We must also add that the alternative of Vxx would be isovaline, which is a C(α)-tetrasubstituted, helicogenic, chiral α-amino acid, and would have very different effect on peptide folding and possibly on the bioactivity, if present in the sequence. In peptaibols, it can be found as either L- or D- configuration or both, although it may not affect the overall screw sense of the entire helix by a significant level [14]. It also has higher helical propensity than valine and may result in stricter α-helical conformations.

### 3.2 Peptaibol bioactivity and MIC test results

The effects of the peptaibol mixtures on nine commonly known Gram-positive and Gram-negative bacteria were investigated to examine their expressed bioactivity. Plant pathogenic bacteria (*Clavibacter michiganensis*, *Rhodococcus fascians*, *Rhizobium radiobacter*, *Xanthomonas campestris*, *Erwinia amylovora* and *Pantoea ananatis*) were specifically selected to investigate the possible future practical application of the peptaibol mixtures. Gram-negative bacteria have previously been reported to be mostly unaffected by the application of peptaibols, however, they may reduce the virulence abilities of Gram-negative bacteria, such as biofilm-formation [56]. Primarily, the peptaibol mixtures inhibited the Gram-positive bacteria, however, extracts from *T. longibrachiatum* f. *bissettii* had an inhibitory effect on the Gram-negative *Rhizobium radiobacter* strain as well at the concentration of 1.25 mg ml^−1^ (Table 6). Furthermore, the peptaibol mixtures from this *Trichoderma* strain also exerted a strong inhibitory effect against Gram-positive bacteria with MIC values of 0.156 mg ml^−1^ and 0.625 mg ml^−1^. *T. longibrachiatum* SZMC 1775, which also produces group A peptaibols, showed a weaker activity against bacteria compared to the clinical isolate *T. longibrachiatum* f. *bissettii*, and was inactive against Gram-negative bacteria. The bioactivity of peptaibol extracts was tested against additional Gram-negative bacteria (*Xanthomonas campestris* SZMC 6182, *Erwinia amylovora* SZMC 21402, *Escherichia coli* SZMC 0582*, Pantoea ananatis* SZMC 6205 J) at a concentration of 10 mg ml^−1^, however, the extracts did not inhibit the growth of these bacteria (Table 6).

**Table 6:** MIC (mg ml^−1^) values calculated for the examined *Trichoderma* strains against commonly known Gram-positive and Gram-negative bacteria.

		Gram-positive				Gram-negative
	*MIC (mg ml*^*−1*^*)*	***Micrococcus luteus*****SZMC 0197**	***Bacillus cereus*****SZMC 23292**	***Clavibacter michiganensis*****SZMC 0016**	***Rhodococcus fascians*****SZMC 21247**	***Rhizobium radiobacter*****SZMC 21407**

**Group A**	***T. longibrachiatum*****f.*****bissettii*****SZMC 12546**	0.156	0.156	0.156	0.625	1.25
	***T. longibrachiatum*****SZMC 1775**	2.5	1.25	0.625	5	-
**Group B**	***T. reesei*****SZMC 22614**	0.625	0.312	0.312	2.5	-
	***T. reesei*****SZMC 22616**	1.25	1.25	1.25	1.25	-
	***T. saturnisporum*****SZMC 22606**	2.5	0.625	0.312	0.312	-
	***T. effusum*****SZMC 22611**	2.5	1.25	1.25	0.312	-

The *T. reesei* SZMC 22614 strain, which has similar peptaibol composition as its mutant *T. reesei* SZMC 22616, exerted higher bioactivity against the *M. luteus*, *B. cereus* and *C. michiganensis* strains (Table 6). Strains *T. saturnisporum* SZMC 22606 and *T. effusum* SZMC 22611 were less effective against *M. luteus* and *B. cereus*. *T. saturnisporum* SZMC 22606 inhibited the *C. michiganensis* and *R. fascians* strains at the concentration of 0.312 mg ml^−1^ while *T. effusum* SZMC 22611 only inhibited *R. fascians* strains at the concentration of 0.312 mg ml^−1^. Most of the peptaibol extracts have a strong inhibitory effect on *C. michiganensis*, while *R. fascians* did not show significant sensitivity, except for the peptaibol extracts from *T. saturnisporum* and *T. effusum*. Numerous *Rhodococcus* species are able to produce biosurfactant compounds that may impair the penetration into the cell wall and the ion-channel forming ability of peptaibols [69], which may explain the low sensitivity to peptaibol treatment.

### 3.3 Reweighted free energy landscape (FEL) plots derived from dihedral-angle principal component analysis: most-likely structures of studied peptaibols revealed from aMD simulations

To understand the dynamic process of peptide folding, we must elucidate the various intermediate ensemble states that occur in a back-and-forth fashion which eventually result in the native folded state. These intermediate metastable states of a peptide are characterized by elevated energy levels when mapped on a potential energy surface of the system, while the ground states or native-like conformations of the peptide will be found on the energy minima. It is interesting to observe how the folding of a peptide evolves from metastable to ground energy conformations by overcoming energy barriers and jumping to the native energy basin. Sometimes, the system can jump multiple times between different energy minimum basins, indicating that the peptide under study has a very dynamic native state and can be defined as an ensemble instead of one rigid structure [41]. The results of all simulations were analyzed by principal component analysis (PCA) which reduces the dimensionality of the obtained data and creates reweighted free-energy landscapes, thereby, revealing the intermediate states and their path to achieve the final folded state [40]. We specifically employed the dihedral-angle based PCA (dPCA) [2] to only include internal motions (defined by ϕ, ψ dihedral angles) for the peptide folding process. The free energy landscape based on internal motions projected along the first two principal components PC 1 and PC 2 using μ(q_1_, q_2_) = −k_B_T lnP(q_1_, q_2_) provides accurate results of the minimum energy wells and barriers between them.

Different conformations of peptaibols obtained during the simulations were clustered by identifying the isolated peaks in a three- or five-dimensional density map obtained from the trajectory’s principal component distribution. Each point on the FEL plot signifies all conformations that have PC values closest to that point. All peaks with density higher than a given threshold are selected which correspond to a distinct cluster. The darkest violet regions on the FEL map show the lowest energy conformation clusters, which denote the low energy/native states of a given peptaibol. These FEL plots make the first part of all the figures while the corresponding cluster information is depicted in the second part. Each conformational cluster is represented with a different colour and shows its occurrence along the simulation run. This provides the “path” of peptide folding where the conformation evolves from less folded metastable states to more folded ground energy states. There could be more than one ground energy state or native-like state observed for peptaibols indicating towards their dynamic nature. In our understanding, this dynamicity of conformational transition may help peptaibols to transform corresponding to different environments in which they are placed.

#### 3.3.1 Folding dynamics of Pept-A-IVa and Pept-B-IXa

**Fig. 2:**
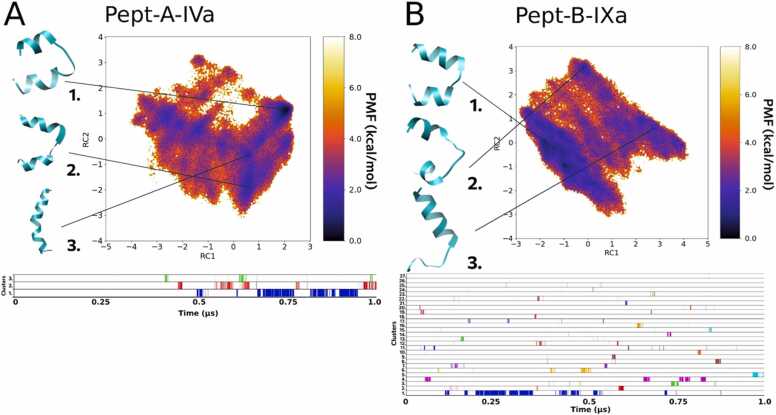
Reweighted free energy landscape plots of (A) Pept-A-IVa (3 clusters) and (B) Pept-B-IXa (27 clusters) along with diagrammatic representation of cluster distribution along the simulation trajectory.

The folding process of the group A peptide Pept-A-IVa produced in the highest amount by both *T. longibrachiatum* strains resulted in three main clusters that were obtained in the second half of the 1 µs long aMD simulation as shown in Fig. 2A. Cluster 1 represented with a large backbone curvature and a shorter N-terminus is the most populated cluster that appears for 15.6% of the total trajectory, while cluster 2 – which also presents with a large backbone curvature but with a longer N-terminal folding – appears for 1.8% of the whole trajectory. The 3rd cluster, however, presented with a highly folded linear backbone (most likely to be the native conformation) appears only for 0.5% of the total trajectory (Table 7). Cluster 1 forms the lowest energy minimum on the FEL plot, while clusters 2 and 3 require an energy „jump” to achieve the highly folded conformations. It can be explained by the fact that these peptides are amphipathic in nature and were simulated in water, and thus, the hydrophobic face of these peptides folds in a manner to hide on the concave side of the backbone curve. That is why it requires an energy boost to achieve the linear conformation in an aqueous environment. From the cluster plot it can be seen that clusters 2 and 3 can readily interchange between the two conformations without requirement of an additional energy cost. This interconversion between the bent and linear forms (rotation along the Aib-Pro kink of the peptides) lies under ∼ 2 kcal/mol and was observed multiple times during the simulations as also reported for alamethicin in a previous study [64]. Some previous studies [23], [50] have pointed towards a possible dynamic equilibrium that exists between the two conformations, and may provide a switch for voltage gating in the formation of ion channels across bilayer membranes.

**Table 7:** Percentage occurrence of each cluster (in frames of simulation) for every sequence.

(Out of 500000 frames)	Cluster 1	%	Cluster 2	%	Cluster 3	%
PA4A	78248	15.6496	9473	1.8946	2950	0.59
PB9A	111751	22.3502	5859	1.1718	23693	4.7386
PA16A	25651	5.1302	34019	6.8038	35953	7.1906
PB16	159963	31.9926	7172	1.4344	9650	1.93
PA25B	58324	11.6648	52182	10.4364	42007	8.4014
PB27	91004	18.2008	948	0.1896	1176	0.2352
PA19A	32293	6.4586	8099	1.6198	20047	4.0094
PB14B	61300	12.26	15028	3.0056	12541	2.5082
PA6A	29900	5.98	20876	4.1752	25392	5.0784
PB14A	117674	23.5348	10624	2.1248	29702	5.9404
PA22B	98251	19.6502	42862	8.5724	17563	3.5126
PB20	171846	34.3692	7970	1.594	3276	0.6552

In comparison, the folding process of the analogous sequence Pept-B-IXa, in which the R12 Leu residue (group A) is replaced with an Aib residue, resulted in a total of 27 distinct clusters, out of which we will focus on the top three most populated ones. The most populated cluster 1 that lies in the lowest energy minimum and occurs for 22.3% of the whole trajectory has a folded helical conformation but shows a hairpin-like backbone bend. Cluster 2, which occurs only for 1.1% of the total trajectory, is represented by an incompletely folded structure, while cluster 3, which occurs for 4.7%, does not show C-terminus folding (Table 7). A linear helical conformation was not observed in the case of Pept-B-IXa (Fig. 2B). The FEL plot describes unrestrictive dynamics where many energy minima lie close to each other, and the different conformations can show easy inter-conversion and loss-of-folding, which signifies an unstable structural configuration.

#### 3.3.2 Folding dynamics of Pept-A-XVIa and Pept-B-XVI

**Fig. 3:**
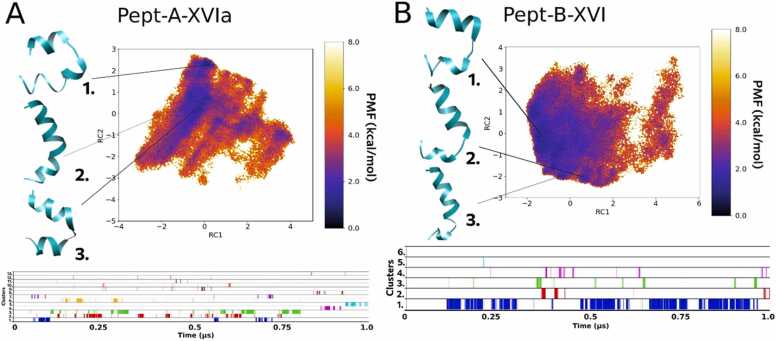
Reweighted free energy landscape plots of (A) Pept-A-XVIa (13 clusters) and (B) Pept-B-XVI (6 clusters) along with diagrammatic representation of cluster distribution along the simulation trajectory.

The peptaibol produced in the second highest amount by both *T. longibrachiatum* strains (group A), Pept-A-XVIa, shows a similar folding process and representative conformations as observed previously for Pept-A-IVa, where these two sequences differ by the presence of an Aib residue at position R6 in the former and Ala in the latter (Table 2). Out of the 13 distinct clusters obtained, the top 3 clusters were obtained for 5.1%, 6.8% and 7.1% of the total trajectory of 1 µs, respectively (Table 7). As observed before for Pept-A-IVa, the FEL plot of Pept-A-XVIa shows Cluster 1 with a large backbone curvature and shorter N-terminus as the most populated distinct cluster, while clusters 2 (folded linear backbone with a bend) and 3 (large backbone curvature but with a longer N-terminal folding) can readily interchange between the two conformations as they lie close to each other (Fig. 3A). Its closest analogous sequence in group B, Pept-B-XVI also shows unrestrictive dynamics of peptide folding, but the obtained conformations are more folded than observed for Pept-B-IXa. Out of the 6 distinct clusters obtained, the top 3 clusters were obtained for 31.9%, 1.4% and 1.9% of the total trajectory, respectively (Table 7). The backbone kink obtained for representative conformations of clusters 2 and 3 is more pronounced than previously observed for the group B Pept-B-IXa sequence (Fig. 3B).

#### 3.3.3 Folding dynamics of Pept-A-XXVb and Pept-B-XXVII

Pept-A-XXVb in group A (produced only by *T. longibrachiatum* SZMC 1775 in small quantities) is closest to the sequence Pept-B-XXVII which is the peptaibol produced in the highest amount in group B, and they also differ only by the Leu12→Aib12 substitution, but also differ from the rest by the presence of a Leu residue instead of Val in the R9 position and a Val residue in R17 instead of Aib. Even though both Leu and Val residues are branched and hydrophobic, this substitution at R9 may affect the interaction with host membranes. A similar case was studied by Ghosh et al. [26], where Leu was substituted by Val at various positions and a loss of bioactivity and micelle/SDS interaction was reported for the antimicrobial peptide. Okamoto [51] also classified Leu to be a ‘helix-former’ while Val to be a ‘helix-breaker’ using Monte Carlo simulations. Therefore, these sequences with R9 Leu may exhibit higher helicity and foldability. On the other hand, Li and Deber [39] measured the helical propensity of amino acid residues in membrane environments and highlighted an interesting property of β-branched Val and Ile as best ‘helix-promoters’ in membranes while acting as helix-destabilizers in aqueous environments. In the same work, they highlighted the high helical propensity of Ala and Leu measured specifically in aqueous media, while in the SDS media, the propensity shifted in the given order: Ile>Leu>Val>>Ala>Gln>>Ser.

**Fig. 4:**
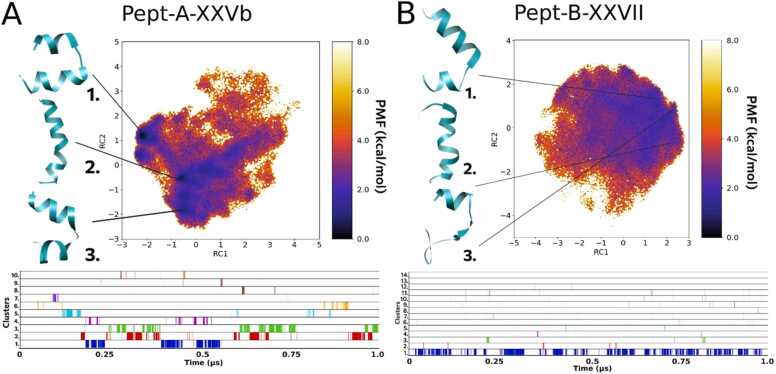
Reweighted free energy landscape plots of (A) Pept-A-XXVb (10 clusters) and (B) Pept-B-XXVII (14 clusters) along with diagrammatic representation of cluster distribution along the simulation trajectory.

The R17 Aib→Val substitution could be considered helix-stabilizing in membrane environments but destabilizing in aqueous environments. Val is a branched amino acid with a bulky side-chain near to the backbone and a strong right-handed screw sense which can restrict the conformations the main-chain can adopt in direct contrast to the properties of achiral Aib residue. The Leu12→Aib12 substitution shows the same result as discussed before, that the presence of Leu shows a restricted folding pattern with more distinguishable energy minima for each representative folded structure. Out of 10 clusters formed in the case of Pept-A-XXVb (Fig. 4A), the top 3 clusters appeared for 11.6%, 10.4% and 8.4% of the times, respectively, while out of 14 clusters for Pept-B-XXVII (Fig. 4B), the top 3 clusters appeared for 18.2%, 0.1% and 0.2% of the times, respectively (Table 7). The top cluster for Pept-B-XXVII shows a bent helical structure with a hinge-like region joining the two helical components, while the 2nd cluster is a misfolded helix and does not occur for a considerable time in the simulation indicating its instability. As Pept-B-XXVII is the most produced peptaibol, this may explain the lower bioactivity of *Trichoderma* strains producing group B peptaibols. Even though Pept-A-XXVb has the same amino acid composition at R9 and R17 positions as in Pept-B-XXVII, with R17 Val most probably bringing helix destabilization, the R12 Leu has the most effect in Pept-A-XXVb helicity as observed in the FEL plot.

#### 3.3.4 Folding dynamics of Pept-A-XIXa and Pept-B-XIVb

**Fig. 5:**
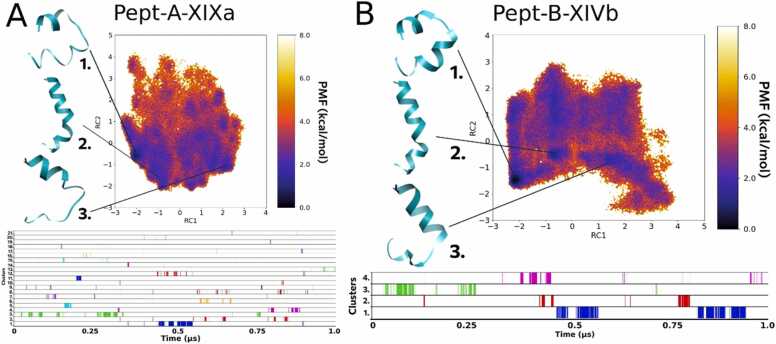
Reweighted free energy landscape plots of (A) Pept-A-XIXa (21 clusters) and (B) Pept-B-XIVb (4 clusters) along with diagrammatic representation of cluster distribution along the simulation trajectory.

On the other hand, FEL observations from Pept-A-XIXa and its closest sequence in group B, Pept-B-XIVb (both produced in lower quantities) revealed that they seem to behave slightly differently than what has been observed for the previous sequence pairs. Both sequences differ from the rest by the presence of an R18 Glu substitution. Simulations from both sequences produced almost identical representative structures for the top 2 most populated clusters. However, the trajectory for Pept-A-XIXa was clustered into 21 clusters, out of which the top 3 clusters were obtained only for 6.4%, 1.6% and 4.0% of the total trajectory (Fig. 5 A), respectively, while Pept-B-XIVb formed only 4 clusters, out of which the top 3 clusters were obtained for 12.2%, 3.0% and 2.5% of the total trajectory (Fig. 5B), respectively (Table 7). Nevertheless, the highly curved conformation of cluster 1 lies in the lowest energy minima in both cases, but the linear, folded cluster 2 conformation of Pept-A-XIXa lies at ∼2 kcal/mol. For Pept-B-XIVb, cluster 2 lies at another distinct low energy minimum closer to ∼0 kcal/mol. In other words, even though a highly folded linear conformation is represented as cluster 2 for both of these peptides, but it is present at an energy minimum (∼0 kcal/mol) or ground energy state for Pept-B-XIVb signifying its stability. For Pept-A-XIXa, cluster 2 conformation lies at a higher energy plateau (metastable state) and therefore, signifies that the linear conformation is not stabilized and prefers the backbone curvature represented by cluster 1.

#### 3.3.5 Folding dynamics of Pept-A-VIa and Pept-B-XIVa

**Fig. 6:**
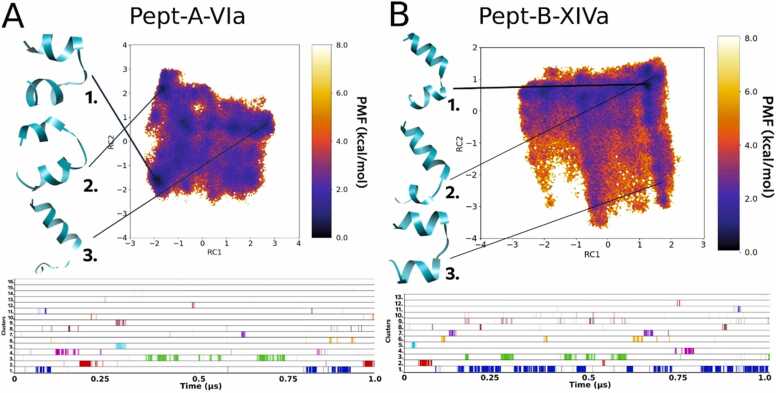
Reweighted free energy landscape plots of (A) Pept-A-VIa (16 clusters) and (B) Pept-B-XIVa (13 clusters) along with diagrammatic representation of cluster distribution along the simulation trajectory.

The other such sequence pair with an R18 Glu residue substitution, Pept-A-VIa and Pept-B-XIVa also differ amongst each other in the R9 position with a Val residue present in the former and replaced by a Leu residue in the latter, along with the usual, R12 Leu→Aib substitution. The conformations of Pept-A-VIa are curved as a hairpin-like helical fold at the center and do not reach any degree of linearity, while the Pept-B-XIVa conformations show highly-folded kink structure bent at the Aib-Pro bond. Furthermore, no distinctive most populated cluster was obtained for the Pept-A-VIa. Both simulations resulted in restrictive dynamics with a total of 16 clusters for Pept-A-VIa, out of which 5.9%, 4.1% and 5.0% are the times of the top 3 clusters appearing in the whole simulation (Fig. 6A). Out of a total of 13 clusters obtained for Pept-B-XIVa, the top 3 clusters appear for 23.5%, 2.1% and 5.9% of the simulation time (Fig. 6B, Table 7).

#### 3.3.6 Folding dynamics of Pept-A-XXIIb and Pept-B-XX

**Fig. 7:**
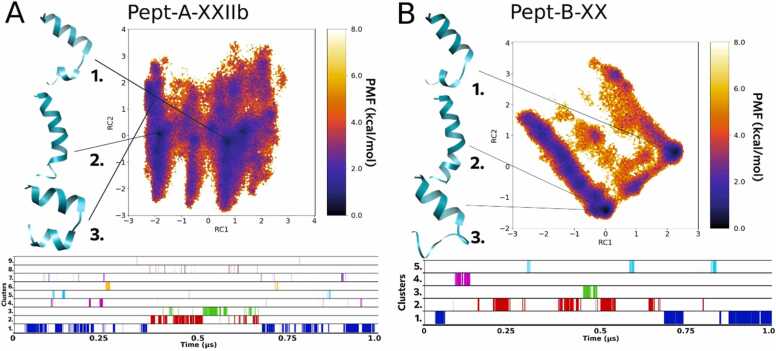
Reweighted free energy landscape plots of (A) Pept-A-XXIIb (9 clusters) and (B) Pept-B-XX (5 clusters) along with diagrammatic representation of cluster distribution along the simulation trajectory.

FEL observations from Pept-A-XXIIb and its closest sequence in group B, Pept-B-XX seem to look very different from each other, even though these sequences just differ in the R12 position along with R9 Val Leu substitution from the other sequences. The Pept-A-XXIIb simulation resulted in a total of 9 clusters with clearly visible energy minima for the first 3 clusters appearing for 19.6%, 8.5% and 3.5% of the simulation time (Fig. 7A). The Pept-B-XX simulation resulted in only 5 clusters, out of which the top three occur for 34.3%, 1.5% and 0.6% of the simulation time, respectively (Table 7, Fig. 7B). In both sequences, the 1st cluster is represented by a folded helix with a kink-like region connecting the two helical folds. The representative structure of cluster 2, which is a linear-folded, helical structure with a bend, is also the same for both sequences, however, it appears for a much longer simulation time in the case of Pept-A-XXIIb than for Pept-B-XX. This observation holds true for all sequences with Leu present in the R9 position instead of Val, and can be explained by higher helical propensity of Leu in proteins [26], [51]. The highly folded, helical conformation either does not appear for a significant time or is not observed at all during the simulation for sequences with R9 Leu, if an Aib is present in the R12 position. In other words, the R9-R12 motif with Leu-Aib-Gly-Leu (as in Pept-A-XXVb and Pept-A-XXIIb) results in more stable folding, but the Leu-Aib-Gly-Aib motif (as in Pept-B-XXVII, Pept-B-XIVa and Pept-B-XX) results in insignificant number of frames or no observation with fully folded helical structures.

### 3.4 Structure-activity relationships observed for the studied peptaibols

In this study, we have attempted to explore the folding dynamics of the peptaibols, *i.e.*, the interconversion of metastable states to native-like conformations on an energy potential. The resultant lowest-energy conformations tell about the most-likely structure of the studied peptaibol. We wanted to see if the substitution (at R12 position) found in these sequence groups can lead to differences in their folding dynamics and if yes, whether it can explain our results with the MIC tests. It is understood that helicity is an important factor of peptaibol bioactivity. Helicity was discussed as a main component of antibacterial activity of synthetic polypeptides containing Leu and Aib by Zikou et al. [71]. We could observe that group A peptaibols (R12 Leu) mostly resulted in fully folded helical structure, which could explain their higher bioactivity. Moreover, based on the FEL plots, we can determine for how long these folded, helical conformations existed during the simulations. If a conformation is more populated during the clustering, it means that it is likely to be stable and not to lose its structure easily. Therefore, not only high helical propensity, but also structural stability points towards higher antibacterial activity of these peptibols.

The peptide Pept-A-IVa from group A produced in the highest amount by *T. longibrachiatum* SZMC 1775 and *T. longibrachiatum* SZMC 12546 – and interestingly, also produced by *T. saturnisporum* along with its usual group B peptaibols – shows a restrained dynamics with only three most populated clusters observed on the dihedral PCA-based free energy landscape plot (Fig. 2). Two main representative conformations were obtained; a highly curved, hairpin-like and the other highly folded, linear conformation with a slight kink at the Aib-Pro bond. In its closest primary structure, Pept-B-IXa (from group B) the R12 Leu residue is substituted by an Aib residue. This sequence is produced in very small amounts by *T. reesei* strains SZMC 22616 and SZMC 22614. The dynamical folding patterns for Pept-B-IXa can be explained by the presence of an extra Aib forming a Gly-Aib-Aib-Pro motif that would allow unrestricted peptide movement as it can easily oscillate between right- and left-handed helical conformations.

In a previous study by our group on 20-residue paracelsins and their 19-residue counterparts, brevicelsins, it was noted that the root-mean-square-fluctuation or atomic fluctuation was higher in sequences containing more Aib residue at the corresponding sequence position [44]. Moreover, owing to the achiral nature of Aib, the screw-sense of these peptides is usually guided by the adjacent amino acid residue [24], and the Gly-Aib-Aib-Pro motif lacks a stable, adjacent, guiding amino acid residue for effective folding in any given direction. The Gly-Leu-Aib-Pro motif, on the other hand, restricts in Pept-A-IVa the possibilities of multiple low energy stable states owing to the β-branched architecture of the Leu side-chain. No fully folded, linear conformation was retrieved for Pept-B-IXa.

The peptaibol produced in the second highest amount in group A, Pept-A-XVIa, and its closest sequence in group B, Pept-B-XVI differ from each other only by the Leu12→Aib12 substitution, but also differ from the previous pair by the presence of an Aib residue in the R6 position. Similar folding behavior could be observed through their dihedral PCA-based FEL plots.

Pept-A-XIXa and its closest sequence in group B, Pept-B-XIVb (both produced in smaller quantities) differ from each other in the R12 position by Leu→Aib, but also differ from the rest of the sequences by the presence of a Glu residue in R18. The folding dynamics seems to be more restrictive for the group B sequence than the group A sequence in contrast to previously observed sequence-pairs. Glutamic acid being polar and carrying a negative charge makes it highly hydrophilic. Pept-A-XIXa and Pept-A-VIa, both with Leu12-Glu18 motif, resulted in unfolded C-terminus and a hairpin-like structure connected by an unfolded loop as their most populated cluster, respectively, indicating the destabilizing effect of this motif. On the other hand, Pept-B-XIVa and Pept-B-XIVb sequences with Aib12-Glu18 motif showed higher foldability and stable dynamics.

Owing to sequence similarity between the Pept-B-XIVa and Pept-B-XIVb sequences with only two substitutions (Ala→Aib in the R6 and Leu→Val in the R9 position), an identical representative structure is obtained, marked as cluster 1 in the former and as cluster 3 in the latter sequence, but no linear structure was observed for Pept-B-XIVa. De Filippis et al. [13] showed through Ala→Aib substitution, that the thermostability and helical folding could be improved for short peptides with such a substitution due to restriction of the available range of polypeptide backbone conformations. In this case, the point seems to stand true that R6 Aib brought more stability and helicity to the folding dynamics of Pept-B-XIVb than Ala in Pept-B-XIVa. Similarly, the other two sequences in group A produced in high amounts, which also differ only in the R6 position by an Ala→Aib substitution, Pept-A-IVa and Pept-A-XVIa; result in a linearly folded conformation, but for the former sequence it appears only for 0.59% of the time, in contrast to the 15% occurrence of a curved structure. On the other hand, for Pept-A-XVIa, the linear conformation appeared for as long as the curved structure. This shows that the linear conformation is more stable in Pept-A-XVIa, which can be explained by the presence of the R6 Aib residue. This way, the stability order could be defined from the strongest to the weakest with the substitutions of amino acids Leu→Aib→Ala, but the effect of their position in the sequence cannot be ruled out and requires further experimentation. Moreover, with the exception of Pept-A-IVa, all other studied peptaibols with Ala at the R6 position did not produce any cluster which could be represented with a linear helical conformation, further strengthening the role of Aib in increasing helicity and providing stability at this position.

The sequences belonging to group B were found to be less bioactive in the MIC tests against the tested strains in comparison to group A sequences. Interestingly, only the extracts from strain *T. longibrachiatum* SZMC 12546, which produces 5 sequences from group A in higher quantity, could inhibit the Gram-negative bacterium *Rhizobium radiobacter*, among the total of five Gram-negative bacterial strains tested. Results from the four Gram-positive bacterial strains tested indicated that both *T*. *longibrachiatum* strains SZMC 1775 and SZMC 12546, which produce peptaibols belonging to group A, show considerable bioactivity. However, extracts of *T. longibrachiatum* SZMC 1775, which produces only 2 sequences in larger quantities from group A, have a slightly weaker effect on the tested microbes. The main difference between the two strains is that the former is an environmental isolate, while the latter is a clinical isolate. The amounts of peptaibol products produced by these two strains are provided in Supplementary Figure 2.

It is interesting to unfold that higher production of some group A peptaibols results in increased efficacy, as observed for clinical isolate *T. longibrachiatum* SZMC 12546. Pept-A-IXa (produced in the 2nd highest amount) and Pept-A-XXIa (produced in the 4th highest amount) are produced in much higher quantities in the clinical isolate *T. longibrachiatum* SZMC 12546 than in *T. longibrachiatum* SZMC 1775, which may be one of the reasons of the enhanced bioactivity of the former strain. These peptides were not studied for their folding dynamics.

The two group B peptaibols produced in the highest amounts are Pept-B-XXVII and Pept-B-XX (near ∼40% of total production each in *T. reesei* SZMC 22614), which together add up to 86.76% of the complete peptaibol production, are also produced by *T. reesei* SZMC 22616, *T. effusum* and *T. saturnisporum*. These two sequences differ only in the R17 Val→Aib substitution, but their folding dynamics show very different patterns. Pept-B-XXVII (Val at R17) seems to be more unstable than Pept-B-XX (Aib at R17) as seen by the lack of discrete energy minimum basins in the FEL plot (Fig. 4B). In other words, the peptide can easily lose its folded conformation, while Pept-B-XX maintained a stable linear conformation during the simulation. Therefore, a Val substitution at position 17 seems to have reduced the flexibility of the C-terminus. This could also explain the comparatively lower bioactivity observed for group B peptaibols, otherwise salvaged by the Pept-B-XX with an R17 Aib residue and a kinked folded structure represented by cluster 2 (Fig. 7B).

**Fig. 8:**
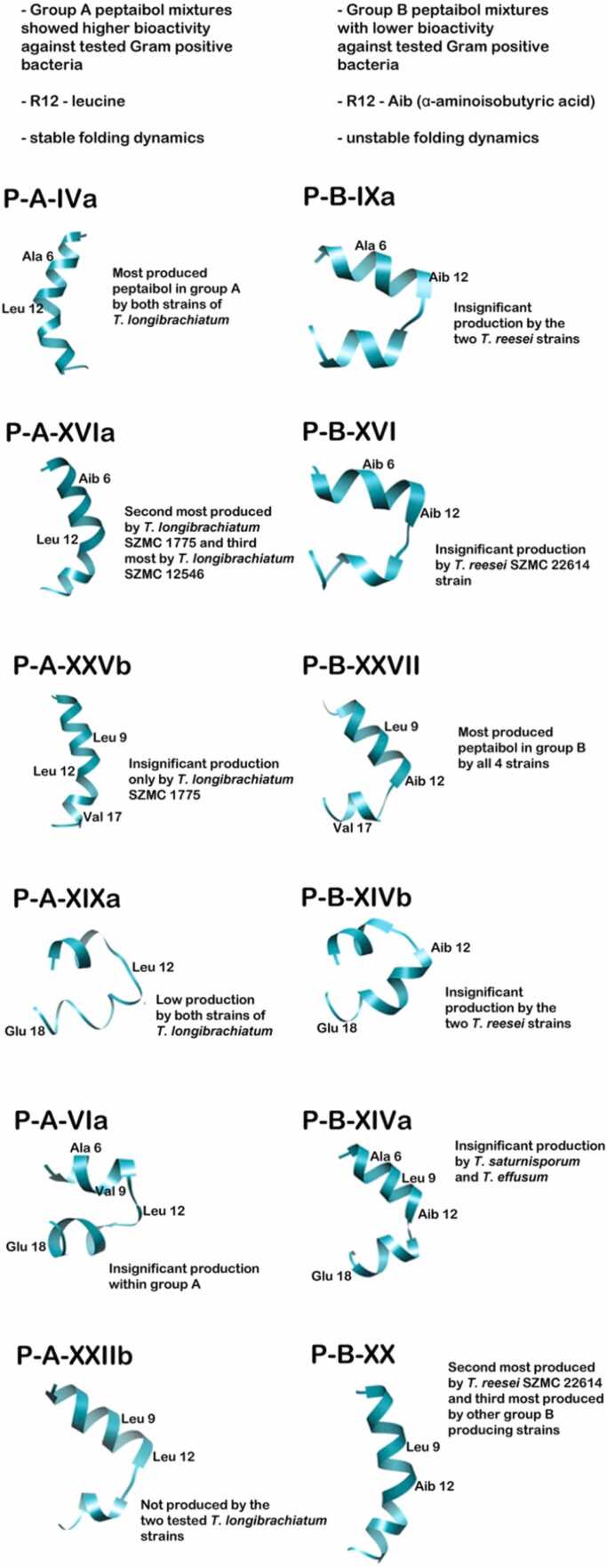
A summary of the main findings of this work. The peptaibols on the left-side belong to group A, while those on the right-side belong to group B.

*T. reesei* SZMC 22614 and *T. saturnisporum* SZMC 22606 also produce group A peptaibols in smaller quantities, along with their usual group B peptaibol production. These two species were found to have better MIC values, especially against *B. cereus* SZMC 23292 and *C. michiganensis* SZMC 0016. *T. reesei* SZMC 22614 (widely known as QM6a) is the wild type which was sampled from the canvas of the US army on the Solomon Islands [54], while *T. reesei* SZMC 22616 (widely known as QM9414) is the mutant of QM9123 (a mutant of QM6) and used for cellulase production in industrial biotecnology [44]. It has been previously discussed that due to the mutations, *T. reesei* SZMC 22616 must have lost its ability to produce group A peptaibols, as they could not be detected from their peptaibol extracts. Marik et al. [44] also suggested that the production of group A peptaibols may be another ancestral trait of the clade Longibrachiatum, while the switch for the production of group B peptaibols might have occurred multiple times and seems to be the result of convergent evolution. This switch from group A to group B has not been fully achieved in certain species, like *T. reesei*, as well as *T. saturnisporum* and *T. konilangbra*, that are also producing some group A compounds in addition to group B peptaibols (Fig. 8).

Furthermore, a direct comparison of the two sequences produced in the highest amount in group A and B, Pept-A-IVa and Pept-B-XXVII, respectively, in terms of their primary structures reveals 4 substitutions (R6: Ala→Aib, R9: Val→Leu, R12: Leu→Aib, and R17: Aib→Val; Table 2). Out of these, the first substitution must bring more stability, as the side-chain physicochemical properties of Aib reduces the potential conformational space of the peptide backbone as discussed earlier. The R9 substitution with Leu must bring more helicity in the aqueous environment as also mentioned earlier. Moreover, it was observed that all sequences with R9 Leu substitution (Pept-B-XXVII, Pept-B-XIVa, Pept-A-XXIIb, Pept-B-XX with the exception of Pept-A-XXVb) result in a bent helical structure with a hinge-like region joining the two helical components as their most populated cluster.

The R12 substitution would be expected to have the same effect as the previous sequence-pairs, with Leu bringing more stability, while Aib introduced an unrestrained dynamics with no clearly marked energy basins for a certain conformation. A study by Conlon et al. [10] outlined the decrease in helicity and hydrophobicity in analogues of the Temporin peptide with Leu9/Ile13/Leu14 → Aib while the Leu6→Aib analogue resulted in decreased cytolytic activity against all tested cell types. Therefore, it has been observed previously that the substitution of Aib from Leu brings decrease in helicity and in some cases in the bioactivity.

As seen above, the R17 substitution in Pept-B-XXVII might make it so rigid to lose the backbone kinked conformation and render it less bioactive. The backbone kink/unfolded hinge-like region has been discussed in case of alamethicin to provide more stability and transmembrane activity. The importance of dynamic helix-bending as a gating mechanism of ion channels has been discussed by Kuo et al. [38] and Miyazawa et al. [45], while Duclohier [18] compared the same for three different peptaibols, alamethicin, trichotoxin and antiamoebin. Cheng and Chang [8] discussed the implication of the more stable kinked form of alamethicin rather than the energetically less stable linear form in voltage-gating. They proposed that linearization of the alamethicin helices from the kinked structure upon increasing electric potential beyond a threshold may be the first event in voltage gating mechanism. The kinks are introduced by helix-breaker residues like proline and glycine in peptaibols. Kaduk et al. [33] showed that substitution of Gly11 and Pro14 in alamethicin did not affect channel formation but reduced conductance levels and significantly reduced lifetimes. Peptides like Pept-B-XXVII may undertake a more folded form within hydrophobic environments like the transmembrane regions but may not remain stable during insertion from the hydrophilic phase to the interior. Therefore, the presence of a restrictive β-branched Leu residue instead of an Aib at the kink region brings higher foldability resulting in higher bioactivity values, but the presence of a hydrophobic β-branched Val residue at the C-terminus instead of an Aib (as in case of Pept-B-XXVII) shows unrestricted dynamics and instability, where the conformation can easily shift into unfolded conformations, as observed in the dPCA free energy landscape plots (Fig. 4B). The first two substitutions at R6 and R9 may bring more stability to Pept-B-XXVII, but the later substitutions at R12 Aib and R17 Val seem to have a greater negative effect on overall foldability, dynamics and bioactivity. Upon comparison with the MIC tests, peptaibols with restricted folding dynamics with the free energy landscapes separated by energy barriers, and with linear, helically folded structures with a slight backbone kink seem to be preferred for higher bioactivity. These characteristics were mostly observed for group A sequences with Leu present at the R12 instead of Aib. It would be interesting to study the structure-activity relationship of these peptaibols using circular dichroism spectroscopy, which can definitely point us towards discrete secondary structural elements found in studied peptaibols in various solvents. Furthermore, their conformational shift observed when placed in transmembrane systems could also be studied to confirm the effects of amino acid substitutions on ionophoric activity.

## 4 Conclusions

In this work, we attempted to draw plausible correlations between the folded, native-like structures of peptaibols and their bioactivities against multiple Gram-positive and Gram-negative bacterial species. These peptaibols are produced as secondary metabolites of the fungal genus *Trichoderma*. The energetics and dynamical folding of these peptides was studied *via* a modern molecular modeling technique known as accelerated molecular dynamics simulation. This study outlined the increased bioactivity for peptaibols belonging to group A which have a Leu residue present in the R12 position instead of an Aib residue like in those belonging to group B. Few other observations were that Ala→Aib substitution at R6 position and Val*→*Leu at R9 may increase helicity and foldability, while R17 Aib→Val substitution introduced backbone rigidity and resultant lower bioactivity. The main conclusions are that the ‘Gly-Leu-Aib-Pro’ motif of group A peptaibols seemed to enhance their bioactivity as outlined by well-defined separate energy basins (restricted folding dynamics) with well-folded helical conformations as the most stable representative structure. On the other hand, the ‘Gly-Aib-Aib-Pro’ motif of group B peptaibols introduced unrestricted folding dynamics, which seemed to be directly correlated with comparatively weak bioactivity values against the tested bacterial strains. All simulations were carried out in an aqueous solvent and the effect of the solvent cannot be ruled out from the observed dynamics. Peptaibols are naturally poorly soluble in water, therefore, further simulations in a more appropriate environment like transmembrane regions may be required and shed light on their mechanisms. Unfortunately, simulations of all-atom representations of membranes are a computationally exhaustive process, but with a progressively changing computational infrastructure it may become a reality soon. Based on our results, the next step will be to simulate the folded structures of highly active peptaibols (obtained through aMD simulation) within transmembrane environments to aid the understanding of their mechanism of action.

## Author contributions

**Dóra Balázs**: Lab experiments and analysis, Performed and analyzed the computational simulations, Formal analysis, Data curation, Writing – original draft Preparation, Visualization; **Tamás Marik**: lab experiments and analysis, Methodology, Formal analysis, Investigation, Writing – original draft preparation, Writing – review & editing; **András Szekeres**: Methodology, Writing – original draft preparation, Writing – review & editing, Supervision; **Csaba Vágvölgyi**: Writing – review & editing, Supervision, Funding acquisition; **László Kredics**: Conceptualization, Writing – review & editing, Supervision, Funding acquisition; **Chetna Tyagi**: Conceptualization, Performed and analyzed the computational simulations, Methodology, Formal analysis, Data curation, Writing – original draft preparation, Writing – review & editing, Visualization, Resources.

## Conflict of interest

The authors declare no conflict of interest.

## References

[1] Agrios G.N. (2005). Plant pathology.

[2] Altis A., Nguyen P.H., Hegger R., Stock G. (2007). Dihedral angle principal component analysis of molecular dynamics simulations. J Chem Phys.

[3] Bissett J. (1991). A revision of the genus *Trichoderma*. II. Infrageneric Classif Can J Bot.

[4] Bissett J., Szakacs G., Nolan C.A., Druzhinina I., Gradinger C., Kubicek C.P. (2003). New species of *Trichoderma* from Asia. Can. J. Bot.

[5] Bobone S., Gerelli Y., De Zotti M., Bocchinfuso G., Farrotti A. (2013). Membrane thickness and the mechanism of action of the short peptaibol trichogin GA IV. Biochim Biophys Acta – Biomembr.

[6] Case D.A., Betz R.M., Cerutti D.S.C., Cheatham T.E. (2016).

[7] Case D.A., Ben-Shalom I.Y., Brozell S.R., Cerutti D.S.C., Cheatham T.E. et al. Proceedings of the AMBER 2018, San Francisco, CA, USA, 2018; Available online: ambermd.org/doc12/Amber18.pdf.

[8] Cheng S.F., Chang D.K. (1999). Proline-induced kink in a helix arises primarily from dihedral angle energy: a molecular dynamics simulation on alamethicin. Chem Phys Lett.

[9] Chugh J.K., Wallace B.A. (2001). Peptaibols: models for ion channels. Biochem Soc Trans.

[10] Conlon J.M., Al-Kharrge R., Ahmed E., Raza H., Galadari S., Condamine E. (2007). Effect of aminoisobutyric acid (Aib) substitutions on the antimicrobial and cytolytic activities of the frog skin peptide, temporin-1DRa. Peptides.

[11] Coutinho T.A., Venter S.N. (2009). Pantoea ananatis: an unconventional plant pathogen. Mol Plant Pathol.

[12] De Cleene M., De Ley J. (1976). The host range of crown gall. Bot. Rev.

[13] De Filippis V., De Antoni F., Frigo M., Polverino de Laureto P., Fontana A. (1998). Enhanced protein thermostability by Ala→Aib replacement. Biochemistry.

[14] De Zotti M., Biondi B., Crisma M., Hjørringgaard C.U., Berg A. (2012). Isovaline in naturally occurring peptides: a nondestructive methodology for configurational assignment. Biopolymers.

[15] De Zotti M., Sella L., Bolzonello A., Gabbatore L., Peggion C. (2020). Targeted amino acid substitutions in a *Trichoderma* peptaibol confer activity against fungal plant pathogens and protect host tissues from *Botrytis cinerea* infection. Int J Mol Sci.

[16] Degenkolb T., Fog Nielsen K., Dieckmann R., Branco-Rocha F., Chaverri P. (2015). Peptaibol, secondary-metabolite, and hydrophobin pattern of commercial biocontrol agents formulated with species of the *Trichoderma harzianum* complex. Chem Biodivers.

[17] Druzhinina I.S., Seidl-Seiboth V., Herrera-Estrella A., Horwitz B.A., Kenerley C.M. (2011). *Trichoderma*: the genomics of opportunistic success. Nat Rev Microbiol.

[18] Duclohier H. (2004). Helical kink and channel behaviour: a comparative study with the peptaibols alamethicin, trichotoxin and antiamoebin. Eur Biophys J.

[19] Dupradeau F.Y., Pigache A., Zaffran T., Savineau C., Lelong R. (2010). The R.E.D. tools: advances in RESP and ESP charge derivation and force field library building. Phys Chem Chem Phys.

[20] Eid M., Rippa S., Castano S., Desbat B., Chopineau J. (2010). Exploring the membrane mechanism of the bioactive peptaibol ampullosporin a using lipid monolayers and supported biomimetic membranes. J Biophys.

[21] Engelberth J., Koch T., Schüler G., Bachmann N., Rechtenbach J. (2001). Ion channel-forming alamethicin is a potent elicitor of volatile biosynthesis and tendril coiling. Cross Talk jasmonate Sali Signal lima bean Plant Physiol.

[22] Essmann U., Perera L., Berkowitz M.L., Darden T., Lee H. (1995). A smooth particle mesh Ewald method. J Chem Phys.

[23] Franklin J.C., Ellena J.F., Jayasinghe S., Kelsh L.P., Cafiso D.S. (1994). Structure of micelle-associated alamethicin from ^1^H NMR. Evidence for conformational heterogeneity in a voltage-gated peptide. Biochemistry.

[24] Freudenberg J., Binder W.H. (2020). Chirality control of screw-sense in Aib-polymers: synthesis and helicity of amino acid functionalized polymers. ACS Macro Lett.

[25] Frisch M.J., Trucks G.W., Schlegel H.B., Scuseria G.E., Robb M.A. (2009).

[26] Ghosh S., Chatterjee S., Satpati P. (2022). Effect of Leu/Val mutation on the energetics of antimicrobial peptide: micelle binding. J Phys Chem B.

[27] Goethals K., Vereecke D., Jaziri M., Van Montagu M., Holsters M. (2001). Leafy gall formation by Rhodococcus fascians. Annu Rev Phytopathol.

[28] Hamelberg D., Mongan J., McCammon J.A. (2004). Accelerated molecular dynamics: a promising and efficient simulation method for biomolecules. J Chem Phys.

[29] Harman G.E., Howell C.R., Viterbo A., Chet I., Lorito M. (2004). *Trichoderma* species — opportunistic, avirulent plant symbionts. Nat Rev Microbiol.

[30] Hatvani L., Manczinger L., Vágvölgyi C., Kredics L., Mukherjee P.K., Horwitz B.A., Singh U.S., Mukherjee M., Schmoll M. (2013). “*Trichoderma* as a human pathogen,” in *Trichoderma - Biology and Applications*.

[31] He K., Ludtke S.J., Heller W.T., Huang H.W. (1996). Mechanism of alamethicin insertion into lipid bilayers. Biophys J.

[32] Huang H.W., Wu Y. (1991). Lipid-alamethicin interactions influence alamethicin orientation. Biophys J.

[33] Kaduk C., Dathe M., Bienert M. (1998). Functional modifications of alamethicin ion channels by substitution of glutamine 7, glycine 11 and proline 14. Biochim Biophys Acta.

[34] Kredics L., Antal Z., Dóczi I., Manczinger L., Kevei F. (2003). Clinical importance of the genus *Trichoderma*. Acta Microbiol Immunol Hung.

[35] Kubicek C.P., Steindorff A.S., Chenthamara K., Manganiello G., Henrissat B. (2019). Evolution and comparative genomics of the most common *Trichoderma* species. BMC Genom.

[36] Kuhls K., Lieckfeldt E., Samuels G.J., Kovacs W., Meyer W. (1996). Molecular evidence that the asexual industrial fungus *Trichoderma reesei* is a clonal derivative of the ascomycete *Hypocrea jecorina*. Proc Natl Acad Sci USA.

[37] Kuhls K., Lieckfeldt E., Samuels G.J., Meyer W., Kubicek C.P. (1997). Revision of *Trichoderma* sect. Longibrachiatum including related teleomorphs based on 100 analysis of ribosomal DNA internal transcribed spacer sequences. Mycologia.

[38] Kuo A., Gulbis J.M., Antcliff J.F. (2003). Crystal structure of the potassium channel KirBac1.1 in the closed state. Science.

[39] Li S.C., Deber C.M. (1994). A measure of helical propensity for amino acids in membrane environments. Nat Struct Biol.

[40] Maisuradze G.G., Liwo A., Scheraga H.A. (2009). Principal component analysis for protein folding dynamics. J Mol Biol.

[41] Maisuradze G.G., Liwo A., Scheraga H.A. (2010). Relation between free energy landscapes of proteins and dynamics. J Chem Theory Comput.

[42] Marik T. (2013). Rapid bioactivity-based pre-screening method for the detection of peptaibiotic-producing *Trichoderma* strains. Acta Biol Szeged.

[43] Marik T., Tyagi C., Racić G., Rakk D., Szekeres A. (2018). New 19-residue peptaibols from *Trichoderma* clade Viride. Microorganisms.

[44] Marik T., Tyagi C., Balázs D., Urbán P., Szepesi A. (2019). Structural diversity and bioactivities of peptaibol compounds from the Longibrachiatum clade of the filamentous fungal genus *Trichoderma*. Front Microbiol.

[45] Miyazawa A., Fujiyoshi Y., Unwin N. (2003). Structure and gating mechanism of the acetylcholine receptor pore. Nature.

[46] Monte E. (2001). Understanding *Trichoderma*: between biotechnology and microbial ecology. Int Microbiol.

[47] Mukherjee P.K., Buensanteai N., Moran-Diez M.E., Druzhinina I.S., Kenerley C.M. (2012). Functional analysis of non-ribosomal peptide synthetases (NRPSs) in *Trichoderma virens* reveals a polyketide synthase (PKS)/NRPS hybrid enzyme involved in the induced systemic resistance response in maize. Microbiology.

[48] Naeimi S., Hatvani L., Marik T., Balázs D., Dóczi I., Cai F., Amaresan N., Sankaranarayanan A., Dwivedi M.K., Druzhinina I.S. (2022). Advances in *Trichoderma* Biology for Agricultural Applications. Fungal Biology.

[49] Neumann N.K., Stoppacher N., Zeilinger S., Degenkolb T., Brückner H. (2015). The peptaibiotics database–a comprehensive online resource. Chem Biodivers.

[50] North C.L., Barranger-Mathys M., Cafiso D.S. (1995). Membrane orientation of the N-terminal segment of alamethicin determined by solid-state ^15^N NMR. Biophys J.

[51] Okamoto Y. (1994). Helix‐forming tendencies of nonpolar amino acids predicted by Monte Carlo simulated annealing. Protein: Struct Funct Bioinfo.

[52] Pierce L.C., Salomon-Ferrer R., Augusto F., de Oliveira C., McCammon J.A. (2012). Routine access to millisecond time scale events with accelerated molecular dynamics. J Chem Theory Comput.

[53] Psurek A., Neusüß C., Degenkolb T., Brückner H., Balaguer E. (2006). Detection of new amino acid sequences of alamethicins F30 by non-aqueous capillary electrophoresis–mass spectrometry. J Pept Sci.

[54] Reese E.T., Levinson H.S., Downing M.H., White W.L. (1950). Quartermaster culture collection. Farlowia.

[55] Richter S., Cormican M.G., Pfaller M.A., Lee C.K., Gingrich R., Rinaldi M.G., Sutton D.A. (1999). Fatal disseminated *Trichoderma longibrachiatum* infection in an adult bone marrow transplant patient: species identification and review of the literature. J Clin Microbiol.

[56] Rogozhin E.A., Sadykova V.S., Baranova A.A., Vasilchenko A.S., Lushpa V.A. (2018). A Novel lipopeptaibol emericellipsin A with antimicrobial and antitumor activity produced by the extremophilic fungus *Emericellopsis alkalina*. Molecules.

[57] Röhrich C.R., Jaklitsch W.M., Voglmayr H., Iversen A., Vilcinskas A. (2014). Front-line defenders of the ecological niche! Screening the structural diversity of peptaibiotics from saprotrophic and fungicolous *Trichoderma*/*Hypocrea* species. Fungal Divers.

[58] Ruiz N., Wielgosz-Collin G., Poirier L., Grovel O., Petit K.E. (2007). New trichobrachins, 11-residue peptaibols from a marine strain of *Trichoderma longibrachiatum*. Peptides.

[59] Samuels G.J., Ismaiel A., Mulaw T.B., Szakacs G., Druzhinina I.S., Kubicek C.P., Jaklitsch W.M. (2012). The Longibrachiatum clade of *Trichoderma*: a revision with new species. Fungal Divers.

[60] Stein T., Vater J., Kruft V., Otto A., Wittmann-Liebold B. (1996). The multiple carrier model of nonribosomal peptide biosynthesis at modular multienzymatic templates. J Biol Chem.

[61] Stoppacher N., Neumann N.K., Burgstaller L., Zeilinger S., Degenkolb T., Brückner H., Schuhmacher R. (2013). The comprehensive peptaibiotics database. Chem Biodivers.

[62] Szekeres A., Leitgeb B., Kredics L., Antal Z., Hatvani L. (2005). Peptaibols and related peptaibiotics of *Trichoderma*. Acta Microbiol Immunol Hung.

[63] Tyagi C., Marik T., Szekeres A., Vágvölgyi C., Kredics L. (2019). Tripleurin XIIc: peptide folding dynamics in aqueous and hydrophobic environment mimic using accelerated molecular dynamics. Molecules.

[64] Tyagi C., Marik T., Vágvölgyi C., Kredics L., Ötvös F. (2019). Accelerated molecular dynamics applied to the peptaibol folding problem. Int J Mol Sci.

[65] van Bohemen A.I., Zalouk-Vergnoux A., Poirier L., Phuong N.N., Inguimbert N., Salah K.B.H. (2016). Development and validation of LC–MS methods for peptaibol quantification in fungal extracts according to their lengths. J Chromatogr B.

[66] Vanquelef E., Simon S., Marquant G., Garcia E., Klimerak G. (2011). F.Y.R.E.D. Server: a web service for deriving RESP and ESP charges and building force field libraries for new molecules and molecular fragments. Nucl Acids Res.

[67] Vicente J.G., Holub E.B. (2013). Xanthomonas campestris pv. campestris (cause of black rot of crucifers) in the genomic era is still a worldwide threat to brassica crops. Mol Plant Pathol.

[68] Yamaguchi H., Kodama H., Osada S., Kato F., Jelokhani-Niaraki M. (2003). Effect of α,α-dialkyl amino acids on the protease resistance of peptides. Biosci Biotechnol Biochem.

[69] Zampolli J., De Giani A., Di Canito A., Sello G., Di Gennaro P. (2022). Identification of a novel biosurfactant with antimicrobial activity produced by *Rhodococcus opacus* R7. Microorganisms.

[70] Zhang Y.B., Zhuang W.Y. (2018). New species of *Trichoderma* in the Harzianum, Longibrachiatum and Viride clades. Phytotaxa.

[71] Zikou S., Koukkou A.I., Mastora P., Sakarellos‐Daitsiotis M., Sakarellos C., Drainas C., Panou‐Pomonis E. (2007). Design and synthesis of cationic Aib‐containing antimicrobial peptides: conformational and biological studies. J Pept Sci: an official publication of the European Peptide. Society.

[72] van der Zwet T., Beer S.V. (1999). Agricultural Information Bulletins.

