# Supplementary material for: Structure-activity correlations for peptaibols obtained from clade Longibrachiatum of Trichoderma: A combined experimental and computational approach
Source: Comput Struct Biotechnol J. 2023 Feb 24;21:1860–73. doi: 10.1016/j.csbj.2023.02.046 (PMC10006723; doi:10.1016/j.csbj.2023.02.046)
Supplement: Supplementary file 1 — Supplementary material. [file mmc1.docx]

# Appendix

## **Appendix A**

Technical constraints, national protected, low-priority and non-focus areas for wind turbine allocation in Switzerland

| **Constraint** | **Excluded areas** | **Data type (*Source*)** |  |
| --- | --- | --- | --- |
| **Technical restrictions** | | |  |
| Steep slopes | Slope >20% | Raster;  Digital elevation model (*200m resolution; Federal office of Topography*) |  |
| Landcover | *glaciers, lakes, wetlands, rivers, rockfields* | Vector;  Swiss Topographic Landscape Model (TLM3D; *Federal office of Topography*) |  |
| Already existing infrastructure | *300m around building zones, airport zones, streets +50m, railways +50m, military areas* | Vector    Swiss harmonized building zones (Federal office for Spatial Development)    Airport perimeters  (*Federal office of Civil Aviation, 2020*)    streets and railways (TLM3D; *Federal office of Topography*)    Military areas (*Federal department of defence, civil protection and sport*) |  |
| **National protected areas without any possibilities to build wind turbines** | | |  |
| Bird protection | National water- and mitigation bird protection zones | Vector;  National water- and mitigation bird protection zones  (*Federal office for the Environment*) |  |
| Wetlands | National protected wetlands (*Germ. Hochmoor, Flachmoore, Moorlandschaften*) | Vector;  Swiss Topographic Landscape Model, Wetland protection perimeter (*Federal office for the Environment)* |  |
| Swiss National Park | *Core perimeter of Swiss National Park* | Vector; Perimeter Swiss National park (*Federal office for the Environment*) |  |
| **Low priority areas** | |  |  |
| *Landscape / nature* | | | |
| UNESCO | UNESCO nature and cultural heritage zones  No wind turbines in the structural and visual area of influence | Vector; UNESCO natural heritage perimeter (*Federal office for the Environment)*    UNESCO cultural heritage *(Federal office of Culture)* |  |
| BLN | national inventory of nature and landscape heritages | Vector; (*Federal office for the Environment)* |  |
| *Habitat and biodiversity protection zones* | | |  |
| Dry meadows | National protected areas of dry meadows  Minimize the negative impact or destruction of sensitive habitats [18a, NHG] | Vector; (*Federal office for the Environment)* |  |
| Floodplains | National protected floodplains serving as habitats for birds | Vector; (*Federal office for the Environment)* |  |
| Hydropower | Areas with an agreement not to use hydropower | Vector; (*Federal office for the Environment)*) |  |
| Buffer of Swiss National Park | Surrounding areas of Swiss National Park | No data available, approximated with a buffer of 2000m around National Park polygons |  |
| *Infrastructure of national interest* | | |  |
| Radars | Meteo and surveillance radar stations with a circular buffer of 5000m | Vector; (*Digitized from topographic map*) |  |
| Airports | Core airport perimeter and obstacle limitation zone around the airports | Vector (*Federal office of Civil Aviation, 2020*) |  |
| Military areas | Military zones and training areas with a additional buffer of 100m | Vector (*Federal department of defence, civil protection and sport*) |  |
| **Non-focus areas** | | |  |
| *Natural and cultural inventories* | | | |
| UNESCO Buffer zones | Buffer zone of UNESCO cultural heritage including structural and visual area of influence | No data available, approximated with 3’000m buffer |  |
| *Habitat and biodiversity protection zones* | | |  |
| Wildlife protection | Swiss wildlife reserves | Vector; (*Federal office for the Environment)* |  |
| UNESCO Biosphere areas | Core areas of UNESCO biosphere reservation | Vector; (*Federal office for the Environment)* |  |
| *Infrastructure of national interest* | | |  |
| Extended buffer zones around radar | Further buffer around meteorological radar stations of 20’000m | Circular buffer of 20’000m |  |

### **Appendix A.1**

Spatial distribution of potential WT for each policy scenario according to table 3.

| 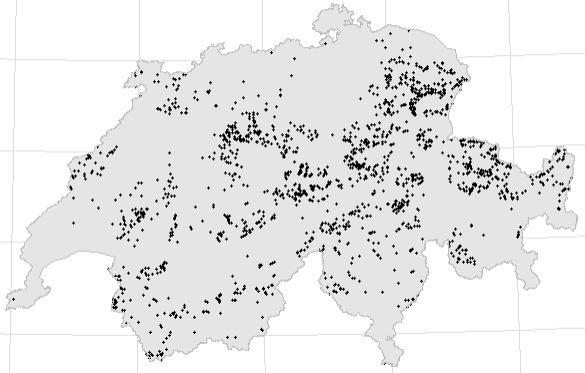  REF | 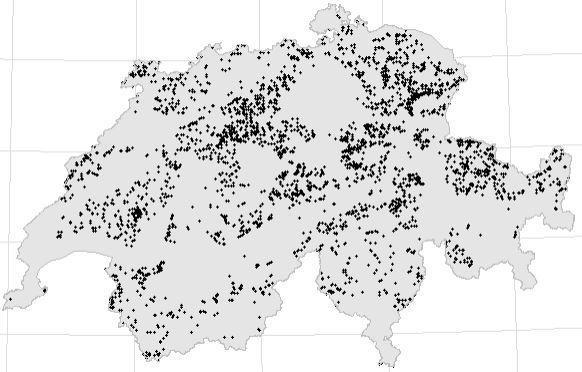  FOR |
| --- | --- |
| 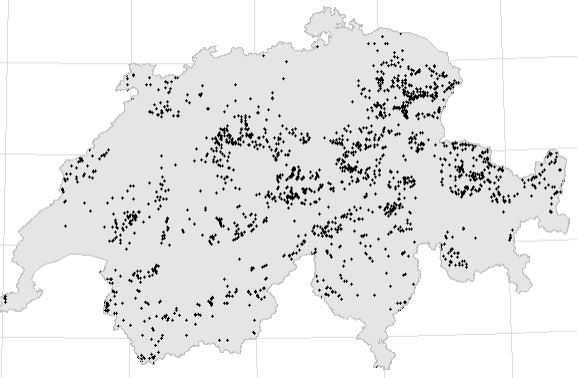  ISOS | 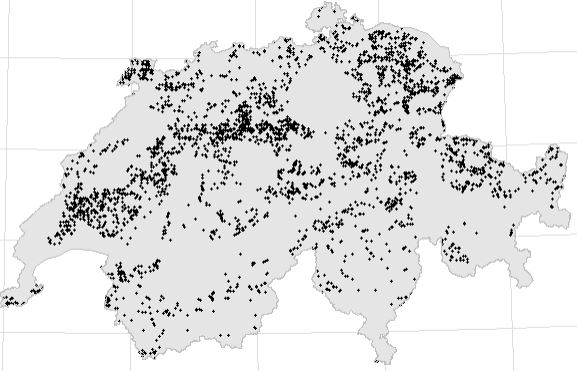  CRF |
|  |  |
| 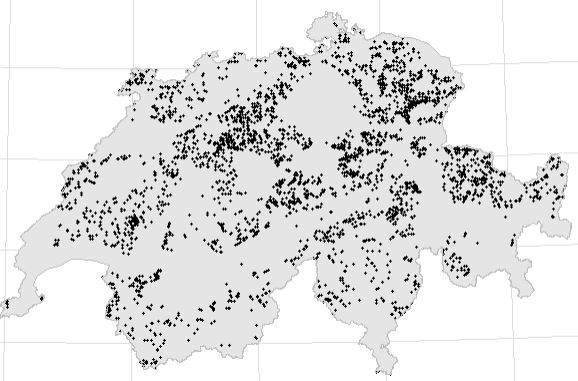  FOR_ISOS | 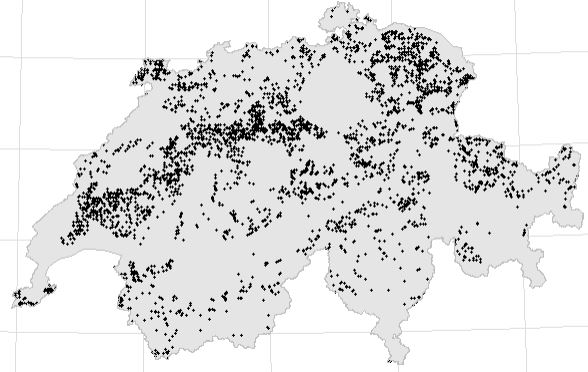  ISOS_CRF |
| 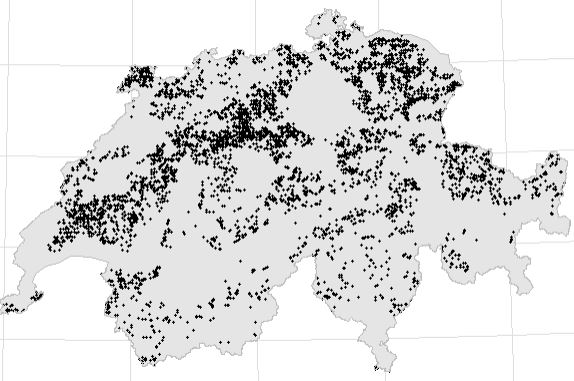  FOR_CRF | 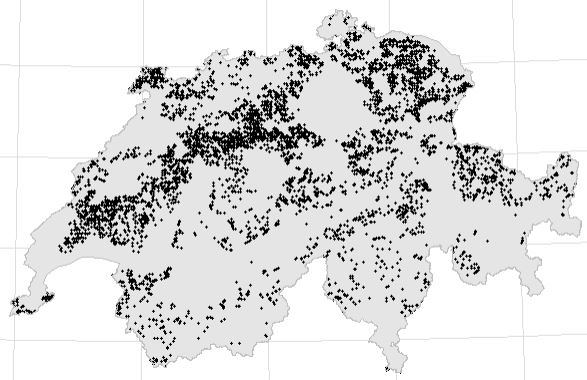  ALL |

## **Appendix B**

**Table B.1** *Icing frequency for Switzerland*

| **Number of days expected for icing** | **Production losses** |
| --- | --- |
| 0-9 | 0 |
| 10-19 | 2.5% - 5% |
| 20-29 | 5% - 7.5% |
| 30-39 | 7.5% - 10% |
| 40-49 | 10% - 12.5% |
| 50-59 | 12.5% - 15% |

The data can be found at Swiss Federal Office of Energy (https://www.bfe.admin.ch/bfe/en/home/supply/statistics-and-geodata/geoinformation/geodata/wind-energy/icing-frequency.html)

## **Appendix C**

Sensitivity Analysis and final optimization setting selection

**Table C.1.** *Four different NSGA-3 optimization runs with different parameter settings (nBits* represents the total number of possible WT for a scenario)

|  | **A** | **B** | **C** | **D** |
| --- | --- | --- | --- | --- |
| Number of generations | 10’000 | 10’000 | 10’000 | 10’000 |
| Population size | 220 | 220 | 220 | 220 |
| Mutation probability | .4 | .2 | .5 | .4 |
| Recombination probability | .7 | .5 | .4 | .7 |
| Probability of Bit Flip mutator | .05 | .05 | .05 | 1/nBITS |

**Table C.2:** Multilinear regression of factors scenarios (SCEN) and different parameters settings (MODEL) on the dependent variables, number of WT (N_WT) clustering (CLUS) and energy density (ENERDENS)


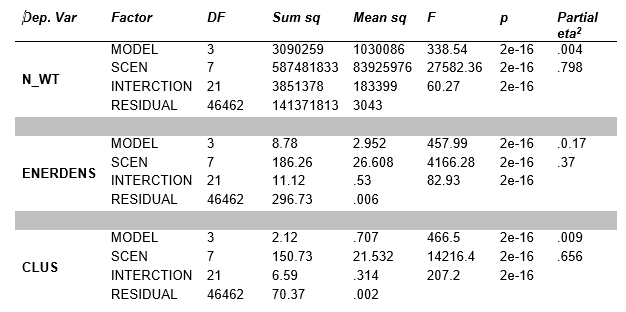


**Table C.3** A * indicates the best NSGA-3 parameter setting for each scenario, selected with the minimum rank sum.


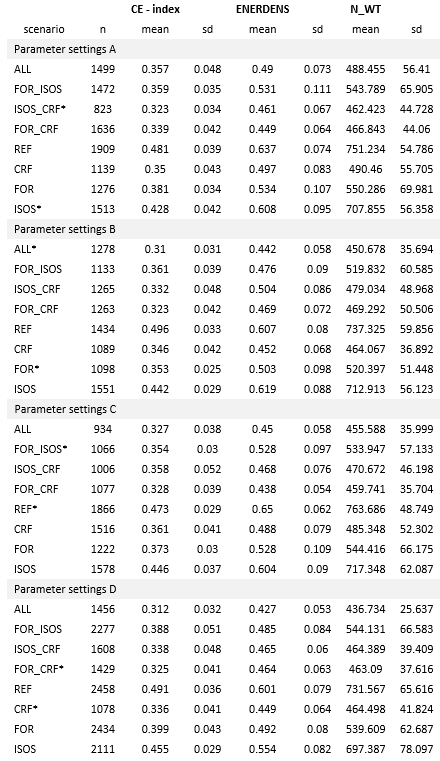


**Appendix E**

Pareto-optimized fitness values separated by optimization goals for each policy scenario

**Figure E.1:** Fitness values of all scenarios for Energy density (a), number of WT (b) and Clark-Evans index c.


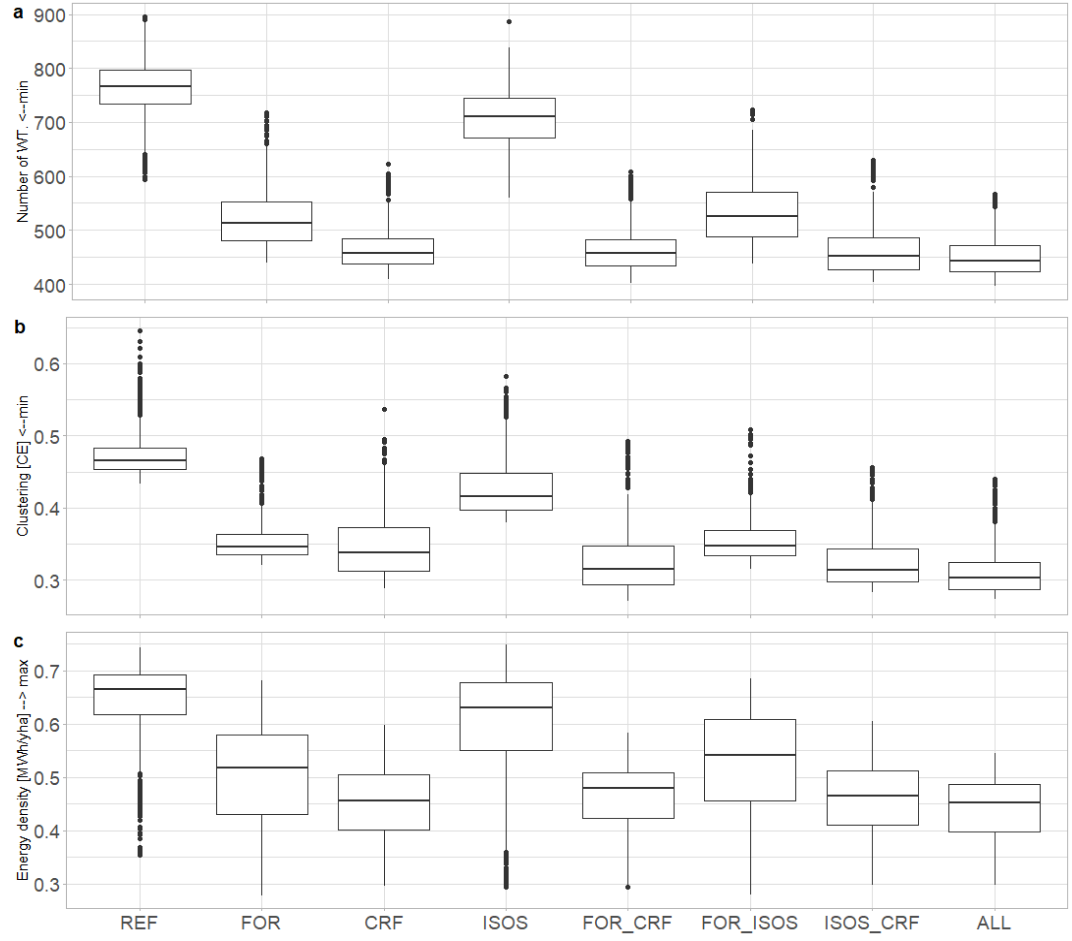


Statistical analysis comparing the fitness values between the scenarios

**Table E.2:** Mann-Whitney U test comparing the means for three goals and between the scenarios.

|  | ***Group 1*** | ***Group 2*** | ***N1*** | ***N2*** | ***W statistic*** | ***P*** | ***Effect size*** |
| --- | --- | --- | --- | --- | --- | --- | --- |
| **N_WT** | REF | FOR | 1866 | 1098 | 3572 | 0 | .83 (large) |
|  |  | ISOS |  | 1513 | 638470 | 0 | .47 (moderate) |
|  |  | CRF |  | 1089 | 23,5 | 0 | .83 (large) |
|  | FOR | FOR_CRF | 1098 | 1429 | 1297309 | 0 | .56 (large) |
|  |  | FOR_ISOS |  | 1066 | 506608 | 0 | .11 (small) |
|  | CRF | CRF_FOR | 1089 | 1429 | 784797 | .77 | Ns |
|  |  | CRF_ISOS |  | 823 | 482096 | .004 | .06 (small) |
|  | ISOS | ISOS_FOR | 1513 | 1066 | 31666 | 0 | .81 (large) |
|  |  | ISOS_CRF |  | 823 | 1243437 | 0 | .82 (large) |
|  | ALL | FOR_CRF | 1278 | 1429 | 723007 | 0 | .18 (small) |
|  |  | FOR_ISOS |  | 1066 | 135287 | 0 | .69 (large) |
|  |  | CRF_ISOS |  | 823 | 42625 | 0 | .11 (small) |
|  | | | | | | | |
| **CLUS** | REF | FOR | 1866 | 1098 | 7460 | 0 | .83 (large) |
|  |  | ISOS |  | 1513 | 500103 | 0 | .55 (large) |
|  |  | CRF |  | 1089 | 27856 | 0 | .81 (large) |
|  | FOR | FOR_CRF | 1098 | 1429 | 1185331 | 0 | .43 (moderate) |
|  |  | FOR_ISOS |  | 1066 | 583773 | .92 | Ns |
|  | CRF | CRF_FOR | 1089 | 1429 | 1022866 | 0 | .26 (small) |
|  |  | CRF_ISOS |  | 823 | 602708 | 0 | .29 (small) |
|  | ISOS | ISOS_FOR | 1513 | 1066 | 94374 | 0 | .75 (large) |
|  |  | ISOS_CRF |  | 823 | 1213101 | 0 | .78 (large) |
|  | ALL | FOR_CRF | 1278 | 1429 | 719008 | 0 | .18 (small) |
|  |  | FOR_ISOS |  | 1066 | 180392 | 0 | .63 (large) |
|  |  | CRF_ISOS |  | 823 | 393033 | 0 | .21 (small) |
|  | | | | | | | |
| **ENERDENS** | REF | FOR | 1866 | 1098 | 188907 | 0 | .68 (large) |
|  |  | ISOS |  | 1513 | 1049754 | 0 | .22 (small) |
|  |  | CRF |  | 1089 | 50638 | 0 | .79 (large) |
|  | FOR | FOR_CRF | 1098 | 1429 | 1004037 | 0 | .24 (small) |
|  |  | FOR_ISOS |  | 1066 | 495060 | 0 | .13 (small) |
|  | CRF | CRF_FOR | 1089 | 1429 | 691970 | 0 | .09 (small) |
|  |  | CRF_ISOS |  | 823 | 417491 | .01 | .05 (small) |
|  | ISOS | ISOS_FOR | 1513 | 1066 | 422228 | 0 | .41 (moderate) |
|  |  | ISOS_CRF |  | 823 | 1106605 | 0 | .64 (large) |
|  | ALL | FOR_CRF | 1278 | 1429 | 709795 | 0 | .19 (small) |
|  |  | FOR_ISOS |  | 1066 | 318008 | 0 | .45 (moderate) |
|  |  | CRF_ISOS |  | 823 | 440916 | 0 | .13 (small) |

## **Appendix F**

**Table F1:** Points of minimal distance to optimal points considering different weights for the planning targets.

| Point number | Weight planning goals | Optimal pts | | | |
| --- | --- | --- | --- | --- | --- |
|  | *N_WT, CLUS, ENERDENS* | Number of WT | Clustering | Energy density | Policy |
| 1 | 33%, 33%, 33% | 550 | .34 | .59 | FOR |
| 2 | 80%, 10%, 10% | 419 | .30 | .35 | CRF |
| 3 | 10%, 80%, 10% | 439 | .29 | .38 | CRF |
| 4 | 10%, 10%, 80% | 819 | .42 | .74 | ISOS |

## **Appendix G**

*Double relaxed policy scenarios*

*Double relaxed policy scenarios*

Spatial distribution

| **FOR_ISOS** *(FOR and ISOS relaxed*  *But not CRF)*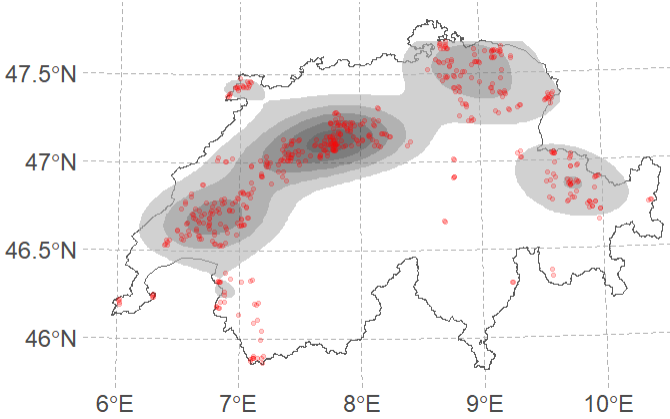  N WT = 524  Mean NNdist = 1’559 m  Mean NDIST_HZ = 1’283 m  Mean NDIST_ROAD= 2’480 m  Clark-Evans Index = .268  Mean elevation [m a.s.l.] = 904 | **CRF_FOR** *(CRF and FOR relaxed but not ISOS)*  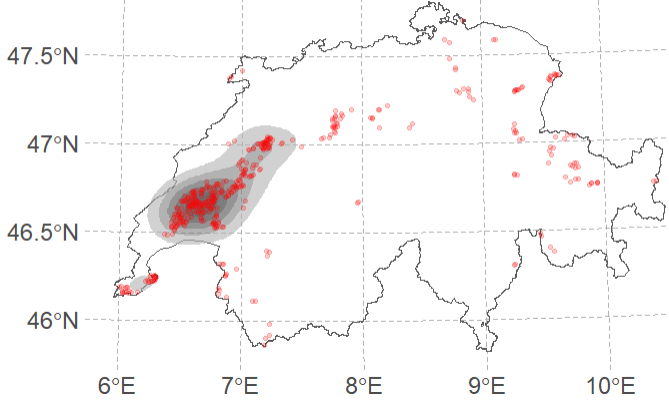  N WT = 454  Mean NNdist = 1’443 m  Mean NDIST_HZ = 1’090 m  Mean NDIST_ROAD = 1’733 m  Clark-Evans Index = .228  Mean elevation [m a.s.l.] = 784 |
| --- | --- |
| **ISOS_CRF** *(ISOS and CRF relaxed but*  *not FOR)*  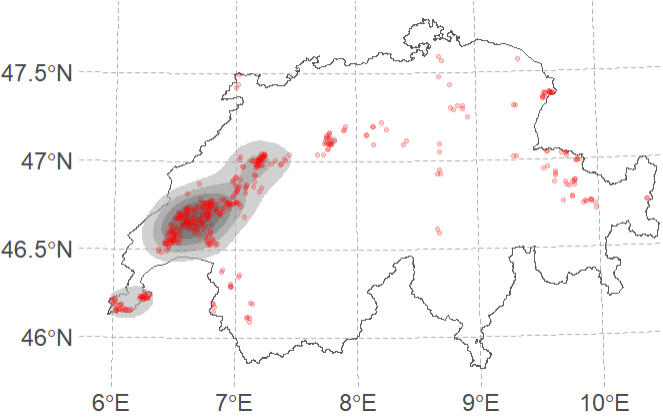  N WT = 446  Mean NNdist = 1’471 m  Mean NDIST_HZ = 1’025 m  Mean NDIST_ROAD =1’647 m  Clark-Evans Index = .255  Mean elevation [m a.s.l.] = 750 | **ALL** *(CRF, FOR and ISOS relaxed)*  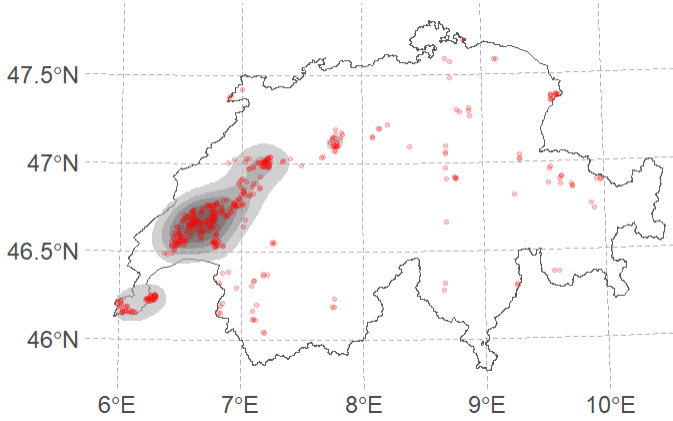N WT = 439  Mean NNdist = 1’577 m  Mean NDIST_HZ = 1’009 m  Mean NDIST_ROAD = 1’763 m  Clark-Evans Index = .27  Mean elevation [m a.s.l.] = 753 |
